# Concurrent inhibition of oncogenic and wild-type RAS-GTP for cancer therapy

**DOI:** 10.1038/s41586-024-07205-6

**Published:** 2024-04-08

**Authors:** Matthew Holderfield, Bianca J. Lee, Jingjing Jiang, Aidan Tomlinson, Kyle J. Seamon, Alessia Mira, Enrico Patrucco, Grace Goodhart, Julien Dilly, Yevgeniy Gindin, Nuntana Dinglasan, Yingyun Wang, Lick Pui Lai, Shurui Cai, Lingyan Jiang, Nicole Nasholm, Nataliya Shifrin, Cristina Blaj, Harshit Shah, James W. Evans, Nilufar Montazer, Oliver Lai, Jade Shi, Ethan Ahler, Elsa Quintana, Stephanie Chang, Anthony Salvador, Abby Marquez, Jim Cregg, Yang Liu, Anthony Milin, Anqi Chen, Tamar Bar Ziv, Dylan Parsons, John E. Knox, Jennifer E. Klomp, Jennifer Roth, Matthew Rees, Melissa Ronan, Antonio Cuevas-Navarro, Feng Hu, Piro Lito, David Santamaria, Andrew J. Aguirre, Andrew M. Waters, Channing J. Der, Chiara Ambrogio, Zhengping Wang, Adrian L. Gill, Elena S. Koltun, Jacqueline A. M. Smith, David Wildes, Mallika Singh

**Affiliations:** 1 Revolution Medicines, Redwood City, CA USA; 2 Department of Molecular Biotechnology and Health Sciences, Molecular Biotechnology Center, University of Torino, Torino, Italy; 3 Department of Surgery, University of Cincinnati, Cincinnati, OH USA; 4 Department of Medical Oncology, Dana-Farber Cancer Institute, Boston, MA USA; 5 Lineberger Comprehensive Cancer Center, University of North Carolina at Chapel Hill, Chapel Hill, NC USA; 6 Broad Institute of MIT and Harvard, Cambridge, MA USA; 7 Human Oncology and Pathogenesis Program, Memorial Sloan Kettering Cancer Center, New York, NY USA; 8 Department of Medicine, Weill Cornell Medical College, New York, NY USA; 9 Molecular Mechanisms of Cancer Program, Centro de Investigación del Cáncer, CSIC–Universidad de Salamanca, Salamanca, Spain; 10 Harvard Medical School, Boston, MA USA; 11 Department of Medicine, Brigham and Women’s Hospital, Boston, MA USA; 12 Department of Pharmacology, University of North Carolina at Chapel Hill, Chapel Hill, NC USA; 13 Department of Cancer Biology, University of Cincinnati, Cincinnati, OH USA

**Keywords:** Targeted therapies, Drug development, Cancer therapeutic resistance

## Abstract

RAS oncogenes (collectively *NRAS*, *HRAS* and especially *KRAS*) are among the most frequently mutated genes in cancer, with common driver mutations occurring at codons 12, 13 and 61^1^. Small molecule inhibitors of the KRAS(G12C) oncoprotein have demonstrated clinical efficacy in patients with multiple cancer types and have led to regulatory approvals for the treatment of non-small cell lung cancer^2,3^. Nevertheless, *KRAS*^G12C^ mutations account for only around 15% of *KRAS*-mutated cancers^4,5^, and there are no approved KRAS inhibitors for the majority of patients with tumours containing other common *KRAS* mutations. Here we describe RMC-7977, a reversible, tri-complex RAS inhibitor with broad-spectrum activity for the active state of both mutant and wild-type KRAS, NRAS and HRAS variants (a RAS(ON) multi-selective inhibitor). Preclinically, RMC-7977 demonstrated potent activity against RAS-addicted tumours carrying various RAS genotypes, particularly against cancer models with *KRAS* codon 12 mutations (*KRAS*^G12X^). Treatment with RMC-7977 led to tumour regression and was well tolerated in diverse RAS-addicted preclinical cancer models. Additionally, RMC-7977 inhibited the growth of *KRAS*^G12C^ cancer models that are resistant to KRAS(G12C) inhibitors owing to restoration of RAS pathway signalling. Thus, RAS(ON) multi-selective inhibitors can target multiple oncogenic and wild-type RAS isoforms and have the potential to treat a wide range of RAS-addicted cancers with high unmet clinical need. A related RAS(ON) multi-selective inhibitor, RMC-6236, is currently under clinical evaluation in patients with *KRAS*-mutant solid tumours (ClinicalTrials.gov identifier: NCT05379985).

## Abstract (web summary)

RMC-7977, a compound that exhibits potent inhibition of the active states of mutant and wild-type KRAS, NRAS and HRAS variants has a strong anti-tumour effect on RAS-addicted tumours and is well tolerated in preclinical models.

## Main

**Extended Data Fig. 1:**
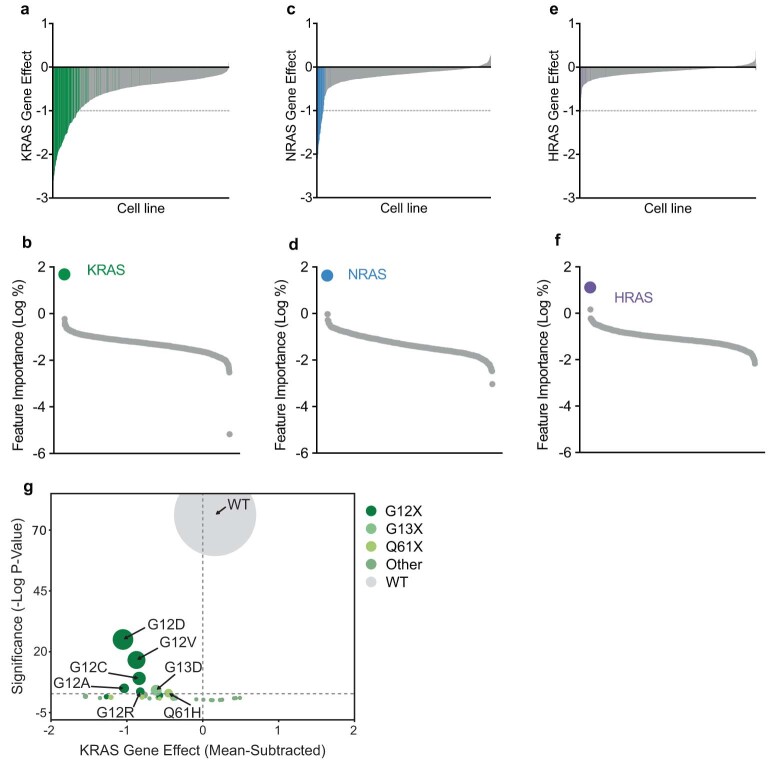
Relationship between RAS gene effect and RAS mutation. **a**,**c**,**e**, Barplots ordered by *KRAS* (**a**), *NRAS* (**c**), or *HRAS* (**e**) CHRONOS score (CRISPR knockout gene effect from https://depmap.org). Each bar represents a cell line. *KRAS*, *NRAS* or *HRAS* mutant cells colored green, blue, or purple respectively. **b**,**d**,**f**, Gene mutation features from trained Random Forest regression model mapping gene mutation status to gene effect. Datapoints represent mutated genes, ordered by importance for the gene effect in each plot. Y-axis indicates the genetic feature importance for *KRAS* (**b**), *NRAS* (**d**), or *HRAS* (**f**) gene effect (see Methods). **g**, Mean KRAS Chronos score for each *KRAS* genotype is shown, with the mean effect score across all cell lines subtracted. P-values were calculated by a two-sided Wilcoxon rank sums test comparing the distribution of genotype effect to the distribution of effect scores outside of that genotype (Bonferonni-corrected p-value of 0.05/31 = 0.0016 indicated by a gray horizontal line, 31 KRAS genotypes tested, point size is proportional to sample size of each genotype, see methods). Source Data

RAS family genes encode small GTPase proteins that regulate cell proliferation in response to growth factor stimuli^1,5^. Cancer-associated *KRAS* mutations are found frequently in non-small cell lung cancer (NSCLC), colorectal cancers (CRC) and pancreatic ductal adenocarcinoma^2^ (PDAC), the three leading causes of cancer deaths in the USA^6^. These activating mutations drive tumour progression by stabilizing the active, GTP-bound (ON) state of RAS proteins and thereby increasing oncogenic flux through downstream effectors^7^. Analysis of CRISPR–Cas9 functional genetic screening data demonstrated that *KRAS*-mutated cancer cell lines are highly sensitive to disruption of the *KRAS* locus (Extended Data Fig. 1a), and *KRAS* mutation status was the only genetic feature that exhibited a significant correlation with *KRAS* dependency (Extended Data Fig. 1b). Similar results were observed for *NRAS* and *HRAS* in *NRAS-* and *HRAS*-mutated lines, respectively (Extended Data Fig. 1c–f). Furthermore, *KRAS* mutations at position 12 are both the most frequent *KRAS* alterations and are associated with the highest degree of *KRAS* dependency compared with other *KRAS* mutations (Extended Data Fig. 1g). This result suggests that although many mutations at *KRAS* codons 12, 13 and 61 have transforming potential^8,9^, not all *KRAS* mutations are associated with equivalent *KRAS* oncogene dependence. Additionally, these data suggest that the *KRAS*^G12X^ mutation is a genetic marker of RAS oncogene addiction and highlight a patient population that may derive a particularly large benefit from a targeted inhibitor of these oncogenic RAS variants.

## RMC-7977 discovery and development

RAS proteins have historically been recalcitrant drug targets^2,3^, although progress in targeting the inactive, GDP-bound state of KRAS(G12C) has resulted in regulatory approvals for two drugs, sotorasib and adagrasib^10,11^. We recently described RMC-4998 and RMC-6291^12^, two covalent tri-complex inhibitors that are designed to target the active state of KRAS(G12C). These macrocyclic compounds were derived from sanglifehrin A, a natural product that binds to cyclophilin A (CYPA) with high affinity^13^. The mechanism of action of these inhibitors is distinct from that of bifunctional immunophilin-binding inhibitors with independent RAS- and CYPA-binding motifs joined by a linker^14^, and instead reflects the binding mechanism of multiple natural products that inspired a paradigm for inhibiting undruggable targets^15,16^. Upon binding CYPA, tri-complex inhibitors remodel the surface of CYPA to create a binary complex with high affinity for active KRAS. Selectivity for KRAS(G12C) is achieved via covalent modification of the reactive thiol group introduced by the oncogenic mutation. The resulting CYPA–compound–KRAS tri-complex sterically blocks KRAS–effector interactions and disrupts downstream signalling.

**Fig. 1:**
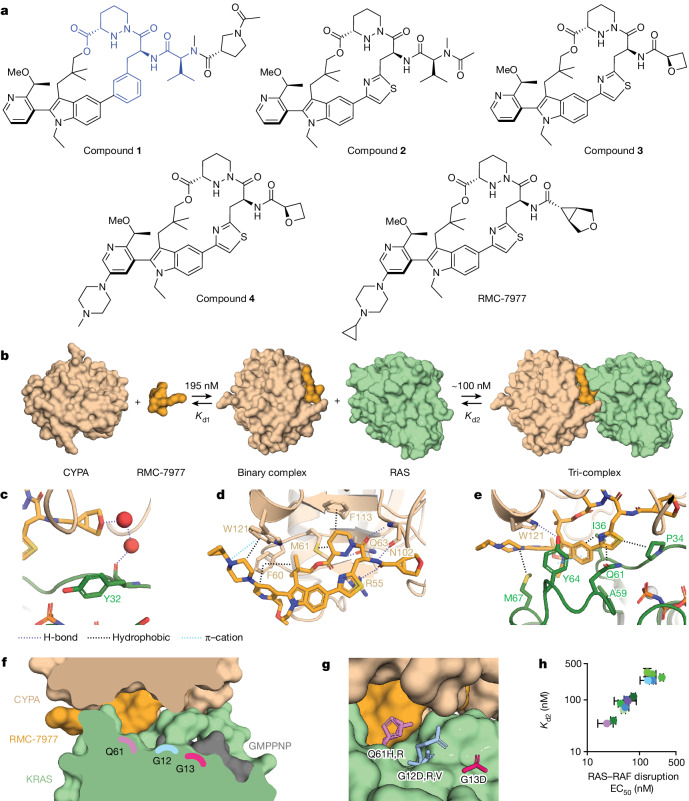
RMC-7977 inhibits the active state of multiple RAS variants. **a**, Compound structures. The CYPA-binding motif of compound **1** is highlighted in blue. **b**, Schematic of tri-complex formation showing reversible binding of RMC-7977 to CYPA (*K*_d1_) and of the binary complex to RAS (*K*_d2_). **c**, A through-water hydrogen bonding network is formed between the ether of RMC-7977 and the carbonyl of RAS Y32 (PDB ID: 8TBM). **d**, CYPA–RMC-7977 binding showing hydrogen bonds, involving R55, the piperazic acid moiety, F113, M61, the geminal dimethyl group, the pyridine and F60. The basic nitrogen of the piperazine forms a cation–π interaction with W121. **e**, Oriented by the hydrogen bond to CYPA W121, RAS Y64 forms π-stacking interactions with the pyridine and indole groups. Apolar sidechains on both SWI and SWII form hydrophobic interactions with RMC-7977. **f**, The tri-complex binding mode creates an open groove between CYPA, KRAS and RMC-7977 along the Q61–G12–G13 axis. **g**, This groove can accommodate the bulky sidechains found in oncogenic mutants, with residues Q61, G12 and G13 measuring 3.5, 7.5, and 9.7 Å, respectively, from RMC-7977 (PDB IDs: 8TBF, 8TBH, 8TBL and 8TBM). **h**, Correlation between *K*_d2_ (determined by surface plasmon resonance (SPR)) and EC_50_ for disruption of RAS–RAF binding in vitro for wild-type and oncogenic RAS mutant proteins. Data are mean ± s.d. of independent experiments (KRAS variants (green): wild-type (WT) KRAS, *n* = 4; KRAS(G12C), KRAS(G12D), KRAS(G12R), KRAS(G12V), KRAS(G13D), *n* = 6; NRAS variants (blue): NRAS(WT), NRAS(Q61L), *n* = 4; NRAS(Q61K), NRAS(Q61R), *n* = 6; HRAS variants (purple): HRAS(WT), *n* = 5; HRAS(G13R), *n* = 6; slope = 0.99, 95% confidence interval: 0.8–1.2, *R*^2^ = 0.99; values also shown in Extended Data Tables 1–3). Source Data

**Extended Data Fig. 2:**
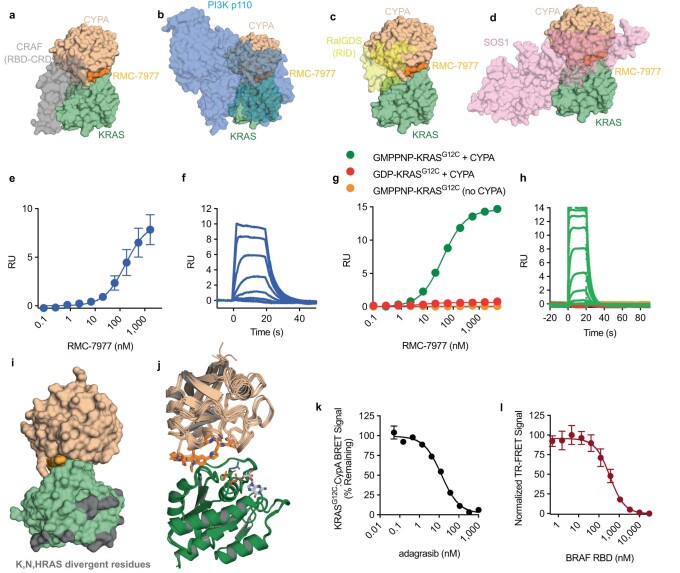
RMC-7977 biophysical and structural characterization. **a**,**b**,**c**,**d**, Superimposition of the CYPA:RMC-7977:KRAS wild-type (WT) tri-complex structure (PDB: 8TBF) with KRAS:CRAF RBD-CRD complex (**a**, PDB: 6XI7), HRAS:PIK3 CD (**b**, PDB: 1HE8), HRAS:RALGDS (**c**, PDB: 1LFD), or KRAS:SOS1 REM-CDC25 (**d**, PDB: 1XD2). Note steric clashes caused by CYPA occupying the Switch I and II motif of KRAS. **e**,**f**, Steady state (**e**) and kinetic sensorgram (**f**) of RMC-7977 and CYPA binding measured by SPR response units (RU). **g**,**h**, Steady state (**g**) and kinetic sensorgram (**h**) of RMC-7977 binding to KRAS^G12C^ measured by SPR. Measurements taken in the presence or absence of CYPA, and GMPPNP or GDP nucleotides. **e**,**g**, Datapoints represent mean ±s.d. of 3 biological replicates. **i**, RAS interacts with CYPA and RMC-7977 through the conserved effector lobe. Isoform divergent residues are distal from the site of interaction, allowing pan-isoform inhibition. **j**, Alignment of wild-type HRAS, wild-type NRAS, and KRAS^G12X^ (A, C, D, R, S, V) mutant tri-complexes shows functionally identical binding modes to the CYPA:RMC-7977 binary complex. 12th position mutant sidechains shown as sticks (PDB: 8TBF, 8TBG, 8TBH, 8TBI, 8TBJ, 8TBK, 8TBL, 8TBM, 8TBN). **k**, Tri-complex formation assay (KRAS^G12C^:RMC-7977:CYPA binding in U2OS cells) in the presence of 100 nM RMC-7977 and the indicated concentration of adagrasib, normalized to the expected min. (100 nM RMC-7977, no adagrasib) and max. (0 nM RMC-7977, no adagrasib) BRET signal. Cells were treated with inhibitor for 4 h. Datapoints represent mean ±s.d. of 6 biological replicates. **l**, Time resolved-FRET between recombinant KRAS^G12V^ and CYPA in the presence of RMC-7977 (50 nM of each) treated with the indicated concentrations of recombinant BRAF RBD, normalized as % of DMSO (EC_50_ = 357 nM, 95% CI = 270-505 nM). n = 3 biological replicates, plotted as mean ±s.d. normalized to control. Source Data

Most RAS oncoproteins with missense mutations are not amenable to selective covalent targeting but could be susceptible to non-covalent inhibition by tri-complex formation with CYPA. In a previous study, we identified compound 2^12^ (referred to in the present Article as compound **1**) (Fig. 1a) with weak, reversible binding to GMPPNP-bound wild-type KRAS and KRAS(G12C). We postulated that we could use structure-guided design to optimize compound 1 to generate a reversible, orally bioavailable inhibitor with broad activity against the active states of multiple RAS variants. Tri-complex formation requires two distinct binding events (Fig. 1b). First, the compound binds to CYPA to form the binary complex (with dissociation constant *K*_d1_). The binary CYPA–compound complex then binds to active RAS (with dissociation constant *K*_d2_) to form a tri-complex structure in which CYPA sterically occludes RAS–effector interactions (Extended Data Fig. 2a–d). Both binding events are essential for tri-complex formation, and we sought to optimize both *K*_d1_ and *K*_d2_ to increase the potency of RAS inhibition, focusing initially on KRAS(G12V) as a representative oncogenic mutant.

Compound **1** contains a CYPA-binding motif (Extended Data Table 1, *K*_d1_ = 862 nM) and forms reversible tri-complexes with KRAS(G12V) (*K*_d2_ = 6,550 nM) that weakly disrupt the binding to the RAS-binding domain (RBD) of BRAF (half-maximal effective concentration (EC_50_) = 4,400 nM). Compound **1** is cell-active, and inhibits RAS pathway activation (phosphorylated ERK (pERK) EC_50_ = 632 nM and proliferation EC_50_ = 965 nM) in Capan-1 cells (PDAC cells with *KRAS*^G12V^ mutation; Extended Data Table 1). Introduction of a thiazole moiety and concomitant scaffold rigidification through rotatable bond reduction and control of hydrogen bond donor count in the side chain yielded compound **2**, with both improved affinity for CYPA (*K*_d1_ = 330 nM) and improved cellular potency (pERK EC_50_ = 31.6 nM; proliferation EC_50_ = 149 nM; Extended Data Table 1). Further reduction of peptidic character resulted in compound **3**, with increased binary complex affinity for KRAS(G12V) (*K*_d2_ = 818 nM) as well as improved oral bioavailability in mice (%*F* = 44), but reduced affinity for CYPA (*K*_d1_ = 6,270 nM) and reduced cellular potency (pERK EC_50_ = 124 nM; proliferation EC_50_ = 615 nM).

To address the reduced CYPA affinity of compound **3**, we introduced a piperazine moiety on the left-hand side of the scaffold to create a cation–π interaction with W121 of CYPA. This modification enhanced not only CYPA binding affinity (compound **4**; *K*_d1_ = 605 nM), but also affinity for KRAS(G12V) (*K*_d2_ = 292 nM) and cellular potency (pERK EC_50_ = 1.94 nM, proliferation EC_50_ = 14.2 nM) (Extended Data Table 1). Structure-guided optimization of a water network-mediated interaction with the Y32 backbone carbonyl of KRAS bound to a GTP analogue (GMPPNP) (Fig. 1c, Protein Data Bank (PDB) ID: 8TBM) resulted in RMC-7977, a potent (*K*_d1_ = 195 nM; *K*_d2_ = 85 nM; pERK EC_50_ = 0.421 nM; proliferation EC_50_ = 2.20 nM) and orally bioavailable (%*F* = 63) RAS(ON) multi-selective inhibitor (Extended Data Fig. 2e–h and Extended Data Table 1). RMC-7977 makes a cation–π interaction between CYPA and the piperazine moiety, and additional hydrophobic and polar interactions are observed, including with the catalytic R55 (Fig. 1d, PDB ID: 8TBM).

Although neither RMC-7977 nor CYPA alone exhibited any measurable binding to GMPPNP–KRAS (Extended Data Fig. 2g,h), RMC-7977 makes multiple π–π and hydrophobic contacts with RAS in the switch I (SWI) and SWII region once the tri-complex is formed (Fig. 1e). All residues in the binding site are identical among HRAS, KRAS and NRAS (Extended Data Fig. 2i), and RMC-7977 is equipotent across these RAS isoforms (Extended Data Table 3). *K*_d2_ measurements for all three wild-type RAS proteins were approximately 100 nM (KRAS *K*_d2_ = 116 nM, NRAS *K*_d2_ =  101 nM and HRAS *K*_d2_ = 94.7 nM). The RMC-7977–CYPA binary complex was highly selective for the active, GMPPNP-bound form of KRAS. No binding was observed for GDP-bound KRAS(G12C) in vitro (Extended Data Fig. 2g,h), and stabilization of GDP-bound KRAS(G12C) with adagrasib treatment prevented KRAS(G12C)–RMC-7977–CYPA tri-complex formation in cells (Extended Data Fig. 2k).

**Extended Data Fig. 3:**
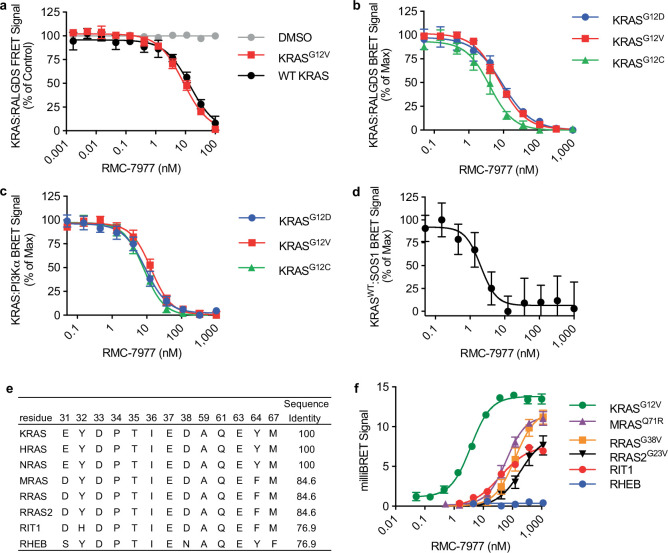
RAS effector inhibition and tri-complex selectivity. **a**, Recombinant KRAS and the RALGDS RID treated with the indicated concentrations of RMC-7977 and CYPA (KRAS^G12V^ IC_50_ = 8 nM, WT KRAS IC_50_ = 14 nM, points represent mean ±s.d. of 4 replicates) **b**,**c**,**d**, Cellular nano-BRET assays for multiple RAS-binding proteins, including full length RALGDS (**b**), full length PI3Kα (**c**) binding to KRAS G12C, G12D, and G12V, and the catalytic domain of SOS1 (**d**) binding to KRAS WT. U2OS cells were treated with RMC-7977 for 1 h. Points represent mean ±s.d. of 6 replicates **e**, Sequence identity analysis of related GTP-ase proteins using KRAS residues positioned within 4 angstroms of RMC-7977 in the tri-complex co-structures as a reference sequence. **f**, Cellular miliBRET signal between CYPA and 5 different small GTPase proteins with moderate to high homology to the KRAS tri-complex interface (RIT1, MRAS, RRAS, RRAS2, and Rheb) treated with RMC-7977. MRAS, RRAS and RRAS2 oncogenic mutants used to induce the active, GTP-bound state. Points represent mean ±s.d. of 6 biological replicates. Source Data

A high-resolution co-crystal structure of RMC-7977 bound to CYPA and GMPPNP-bound KRAS shows a tri-complex with extensive non-covalent interactions and an unoccupied groove containing the common oncogenic residues, G12, G13 and Q61, providing a structural basis for the ability of RMC-7977 to bind these variants (Fig. 1f,g, PDB IDs: 8TBF, 8TBH, 8TBL and 8TBM and unpublished data). Further, RMC-7977 exhibited a consistent binding mode across all KRAS(G12X) mutants tested (Extended Data Fig. 2j, PDB IDs: 8TBF, 8TBG, 8TBH, 8TBI, 8TBJ, 8TBK, 8TBL, 8TBM and 8TBN). *K*_d2_ values for the most common oncogenic RAS variants were all within threefold of those for wild-type proteins (Extended Data Table 2). The ability of tri-complex formation to sterically disrupt effector binding for the various mutants was also measured, revealing a good correlation between the *K*_d2_ measurements and the biochemical EC_50_ values for RAS–RAF disruption (Fig. 1h). Similar potency was also observed for inhibition of KRAS(G12V)–RALGDS (RAS-interacting domain (RID)) binding in vitro (Extended Data Fig. 3a). Coincubation with increasing concentrations of BRAF RBD attenuated tri-complex formation, indicating it is competitive with effector binding (Extended Data Fig. 2l).

**Fig. 2:**
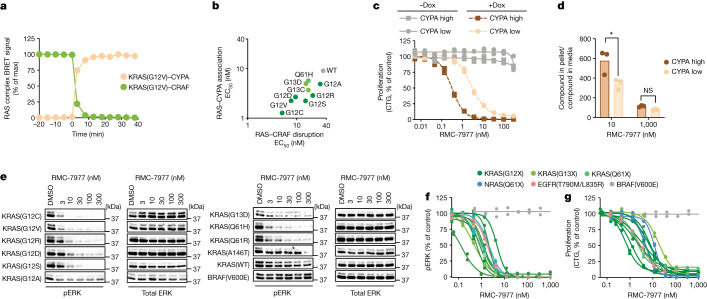
RAS inhibition is CYPA-dependent and active against multiple RAS variants. **a**,**b**, Formation of KRAS–CYPA complexes and disruption of the KRAS(G12V)–CRAF interaction in U2OS cells as a time course after 50 nM RMC-7977 treatment, expressed as % of the maximum (max) signal (**a**), and correlation between potency of RAS–RAF inhibition and formation of the tri-complex for multiple KRAS variants (*R*^2^ = 0.7) (**b**). Data points are single nano-BRET measurements representative of three independent experiments. **c**, Proliferation (measured by CellTiter-Glo (CTG) assay) of NCI-H358 cells with doxycycline (Dox)-inducible expression of low or high CYPA levels treated with RMC-7977 for 120 h. Data points show biological duplicates normalized to vehicle control. Representative data from one of three independent experiments. **d**, Liquid chromatography–mass spectrometry measurements of the ratio of RMC-7977 concentration in CYPA-high and CYPA-low NCI-H358 cells to the concentration in the medium with 1 h compound treatment. Bars depict the mean of biological triplicates from one of two replicate experiments (*P* = 0.012, one-way analysis of variance (ANOVA) with post hoc Tukey’s test). **e**, Western blots of isogenic MEF cells expressing the indicated KRAS variant or BRAF(V600E) and treated with RMC-7977 or DMSO for 24 h. Data are representative of three independent experiments. **f**,**g**, pERK (AlphaLISA) (**f**) and proliferation (CTG assay) (**g**) levels of human cancer cell lines with G12 (Capan-1, SW620, AsPC-1, HPAC, NCI-H358, PSN1 and HUPT3), G13 (HCT 116) or Q61 (Hs 766T) mutant *KRAS*; Q61-mutant *NRAS* (SK-MEL-30 and KU1919); mutant *EGFR* (NCI-H1975); or *BRAF*^V600E^ (A375), treated with RMC-7977 for 4 h. Data points show biological duplicates normalized to vehicle control from 1 of 2–26 independent experiments. Source Data

We used a live-cell nano-bioluminescence resonance energy transfer (BRET) kinetic assay to show that RMC-7977 induced equally rapid (signal half-life (*t*_1/2_) < 5 min; Fig. 2a) association between KRAS and CYPA and dissociation of the CRAF RBD from KRAS, consistent with direct targeting of the active state of RAS in cells accompanied by steric inhibition of protein–protein effector engagement. EC_50_ measurements in this assay were in the single-digit nanomolar range across a panel of wild-type, G12, G13 and Q61-mutant KRAS proteins, and correlated well with EC_50_ values for induced KRAS–CYPA association (Fig. 2b). Similar potencies were observed for inhibition of RALGDS, PI3Kα and SOS1 binding to KRAS(G12C), KRAS(G12V) or KRAS(G12D), as well as SOS1 binding to wild-type KRAS (Extended Data Fig. 3b–d). Tri-complex formation induced by RMC-7977 was also more than tenfold more potent for KRAS than MRAS and other RAS family small GTPase proteins with high sequence identity to KRAS (Extended Data Fig. 3e,f).

**Extended Data Fig. 4:**
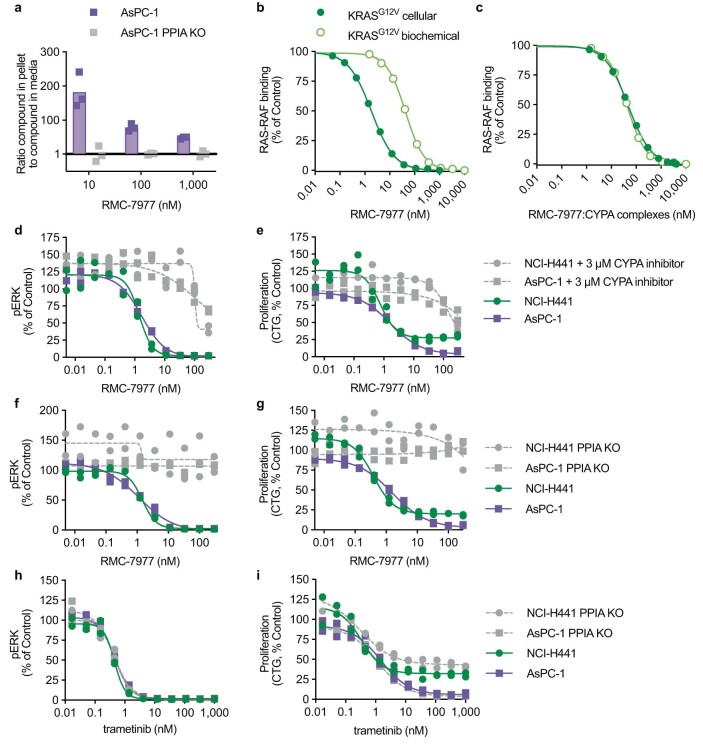
Cellular concentration of CYPA determines the cellular concentration of binary complex. **a**, Ratio of RMC-7977 concentration in cells to concentration in media following exposure of parental or *PPIA* KO AsPC-1 cells to indicated concentrations of RMC-7977 for 1 h as determined by LC/MS bioanalysis. Bars represent mean of the 3 biological replicates shown from one experiment. **b**, RMC-7977 concentration response for biochemical (points are the mean of biological duplicates from one of 6 independent experiments) and cellular (3 independent experiments) nano-BRET KRAS^G12V^-RAF disruption. Data shown are representative of independent replicates. **c**, same data as **b**, with calculated concentration of active binary complexes. Correction is based on equation 1: $\left[{binary\; complex}\right]=\frac{{\left[{CYPA}\right]}_{{cell}}\ast {\left[{RMC}-7977\right]}_{{unbound}}}{{K}_{D}1+{\left[{RMC}-7977\right]}_{{unbound}}}$. This calculation assumes the extracellular volume is much greater than the intracellular volume and that the concentration of unbound RMC-7977 ([RMC-7977]_unbound_) equilibrates between intracellular and extracellular space. A value of 5 µM was used for the cellular CYPA concentration ([CYPA]_cell_). No adjustment was applied to the biochemical data because under experimental conditions >99% of RMC-7977 is bound to CYPA. **d**-**i**, pERK levels (MSD) (**d**,**f**,**h**) and cell proliferation (CTG) (**e**,**g**,**i**) in AsPC-1 and NCI-H441 cells treated with RMC-7977 or trametinib for 4 h. RMC-7977 co-treatment with a CYPA inhibitor^12^ (**d**,**e**) or *PPIA* KO (**f**,**g**) rescued pERK and proliferation in RMC-7977 treated cells, but did not affect response to trametinib (**h,i**). Representative data from one of 2 (NCI-H441) or 3 (AsPC-1) independent experiments are shown. Source Data

The cellular potencies for KRAS–CYPA association were approximately 5 to 50 times higher than the corresponding biochemical *K*_d2_ measurements (Extended Data Tables 1 and 2). An increase in cellular potency compared with biochemical potency is expected based on the tri-complex mechanism of action, in which binding to abundant CYPA drives high intracellular concentrations of CYPA–RMC-7977 binary complexes, as evidenced by accumulation of RMC-7977 in a CYPA-dependent manner in AsPC-1 cells (Extended Data Fig. 4a). Furthermore, biochemical and cellular potencies are similar when adjusted to reflect the estimated intracellular concentration of binary complexes formed in cells (Extended Data Fig. 4b,c).

**Extended Data Fig. 5:**
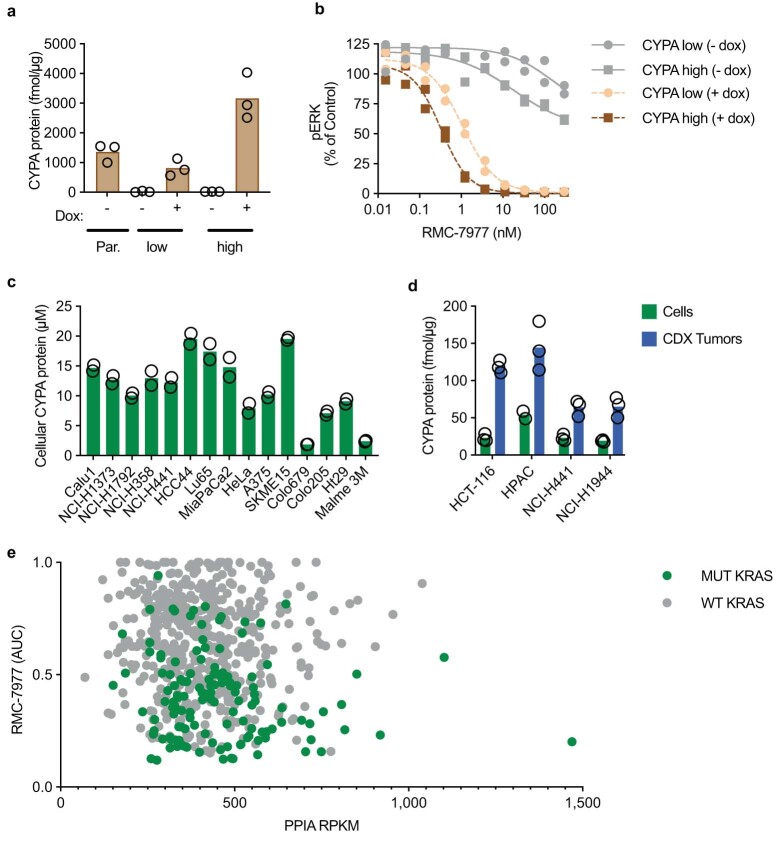
CYPA is required for cellular activity. **a**, Cellular CYPA protein levels determined by parallel reaction monitoring (PRM) mass spectrometry in NCI-H358 cells harboring doxycycline-inducible expression of low or high CYPA in the absence or presence of doxycycline (0.1 µg/ml) for 120 h. Bars indicate the mean of 3 biological replicates per group from one experiment. **b**, pERK levels (MSD) of CYPA high and CYPA low cells treated with RMC-7977 in the absence or presence of doxycycline (0.1 µg/ml) for 120 h. Datapoints show biological duplicates normalized to vehicle control. Representative data shown from one of 3 independent experiments. **c**, Cellular CYPA protein concentration in a panel of cell lines determined by PRM. Data 2 stable isotope labeled peptide standards (SIS) was averaged for each replicate. Intracellular µM concentration estimated assuming cell volume of 2 pl. Bars indicate the mean of biological duplicates. **d**, CYPA protein expression in cells (green bars) and corresponding xenograft tumours (blue bars) determined by PRM. 5 SIS peptides were used and data averaged for each biological replicate. Bars indicate the mean of biological duplicates for HPAC cells and triplicates for all others. **e**, PRISM screen, RMC-7977 sensitivity (AUC) by *PPIA* gene expression (RPKM). Each dot represents a cell line (n = 606). Two-sided Pearson correlation = −0.10 (p = 0.011). Source Data

To verify that formation of the CYPA–RMC-7977 binary complex is essential for cellular activity, we used a competitive CYPA inhibitor^17^ or genetically knocked out *PPIA*, the gene that encodes CYPA. These studies confirm that CYPA binding is required for inhibition of RAF–MEK–ERK signalling and proliferation by RMC-7977 in NCI-H441 (*KRAS*^G12V^, NSCLC) and AsPC-1 (*KRAS*^G12D^, PDAC) cells (Extended Data Fig. 4d–g). As a control, disruption of the *PPIA* locus did not affect sensitivity to the MEK1/2-selective inhibitor, trametinib, which does not rely on the tri-complex mechanism of action (Extended Data Fig. 4h,i). We further investigated whether exogenous CYPA expression could restore RMC-7977 sensitivity in NCI-H358 (*KRAS*^G12C^, NSCLC) cells lacking *PPIA*. We investigated two clones expressing either low or high CYPA levels through a doxycycline-inducible promoter (Extended Data Fig. 5a). Inhibition of pERK and proliferation was CYPA-dependent, and CYPA-high cells were threefold and eightfold more sensitive to RMC-7977 inhibition of signalling and cell proliferation, respectively, compared with CYPA-low cells (Fig. 2c and Extended Data Fig. 5b). RMC-7977 accumulation was significantly greater in CYPA-high cells compared with CYPA-low cells treated with 10 nM RMC-7977, with no difference at 1 µM, at which concentration binding to cellular CYPA is estimated to approach saturation (Fig. 2d). Collectively, these observations suggest that the cellular potency of RMC-7977 is dependent on intracellular concentration of binary complexes, driven by intracellular CYPA protein expression. CYPA is highly abundant in cells (median concentration = 12.3 µM) as measured across a panel of 15 cell lines (Extended Data Fig. 5c), and CYPA expression was higher in cell line-derived xenograft (CDX) tumours in vivo compared with the corresponding cells cultured in vitro (Extended Data Fig. 5d). Finally, CYPA is abundantly expressed across cancer types and exhibits low inter-patient variation in expression^12^, suggesting that tumour expression of CYPA is unlikely to be limiting for RMC-7977 potency. Indeed, *PPIA* mRNA expression across a panel of cancer cells did not correlate with sensitivity to RMC-7977 (Extended Data Fig. 5e).

**Extended Data Fig. 6:**
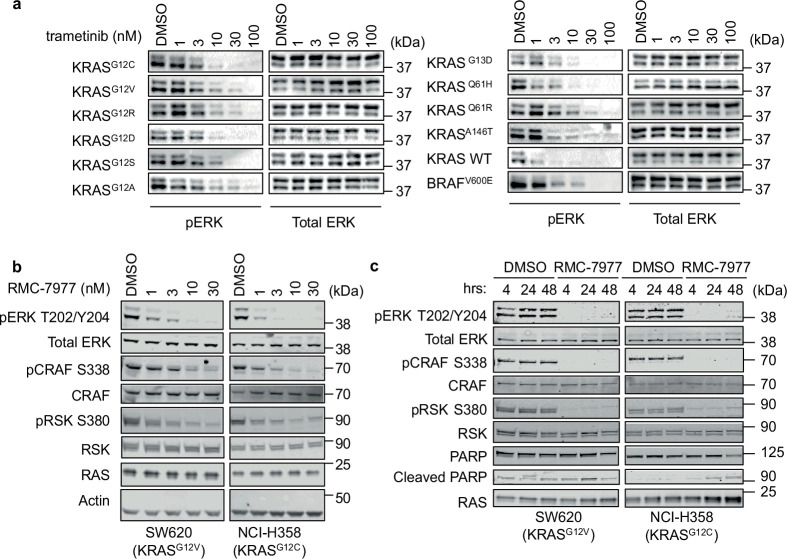
Inhibition of RAS signal transduction pathways. **a**, Western blots of isogenic RAS-less MEF cell lines harboring an exogenous, wild-type (WT) or mutant *KRAS*, or *BRAF*^*V600E*^ transgene, treated with indicated concentrations of trametinib or DMSO control for 24 h. Data shown are representative of 3 independent experiments. **b**,**c**, Western blots of *KRAS* mutated cell lines treated with RMC-7977 at indicated concentrations for 4 h (b), or treated with RMC-7977 (100 nM) or DMSO control for indicated time points. **c**, Data shown are representative of 2 independent experiments. Source Data

RMC-7977 exhibited similar activity for wild-type and mutant RAS variants in biochemical assays, and in the live-cell nano-BRET assay the cellular potency for inhibition of CRAF (RBD) binding to wild-type KRAS was only modestly lower than that for the oncogenic variants. However, many factors can influence the downstream consequences of RAS inhibition in cells. To assess the spectrum of RMC-7977 activity against common KRAS variants in cells, we evaluated a panel of matched mouse embryonic fibroblasts (MEFs) that were null for all three *Ras* genes (RAS-less), in which proliferation was restored with ectopic expression of wild-type or mutationally activated^18^ *KRAS* or *BRAF*^V600E^ (Fig. 2e). RMC-7977 suppressed pERK in all KRAS-expressing cells, but not in BRAF(V600E)-expressing RAS-less MEFs, which lack all RAS proteins and are not RAS-dependent, indicating that pERK suppression is KRAS-dependent. Notably, we observed minor but consistent differences between the various KRAS mutants. pERK suppression by RMC-7977 typically appeared complete across cells expressing various KRAS(G12X) mutants, but consistently reached a plateau in wild-type *KRAS*, *KRAS*^G12A^, *KRAS*^Q61H^, *KRAS*^Q61R^, *KRAS*^G13D^ and *KRAS*^A146T^ cells; by contrast and as expected, trametinib reduced pERK similarly in all cells, including the *BRAF*^V600E^ MEFs (Extended Data Fig. 6a). These differences indicate that *KRAS* genotype could affect the cellular response to direct RAS inhibition, and that the cellular response to RMC-7977 inhibition is not equivalent to that of MEK inhibition.

We also compared RMC-7977 activity in cancer cells harbouring various activating mutations in the RAS pathway, specifically oncogenic variants of *KRAS*, *NRAS*, *EGFR* or *BRAF*. RAS-dependent (*KRAS*, *NRAS* or *EGFR*-mutated) cancer cells treated with RMC-7977 exhibited concentration-dependent inhibition of downstream signalling and proliferation in the low nanomolar range (Fig. 2f,g). In *KRAS*^G12V^ and *KRAS*^G12C^ cells, inhibition of additional markers, including phosphorylation of RAF, ERK and the ERK substrate RSK, was demonstrated (Extended Data Fig. 6b). In these cells, there was evidence of durable pathway suppression and apoptosis induction after 48 h of treatment, indicated by sustained pERK, pCRAF and pRSK suppression and moderately increased PARP cleavage (Extended Data Fig. 6b,c). No inhibition by RMC-7977 was observed in RAS-independent *BRAF*^V600E^-mutant A375 cells (Fig. 2f,g).

## RMC-7977 activity in RAS-addicted cancer

**Fig. 3:**
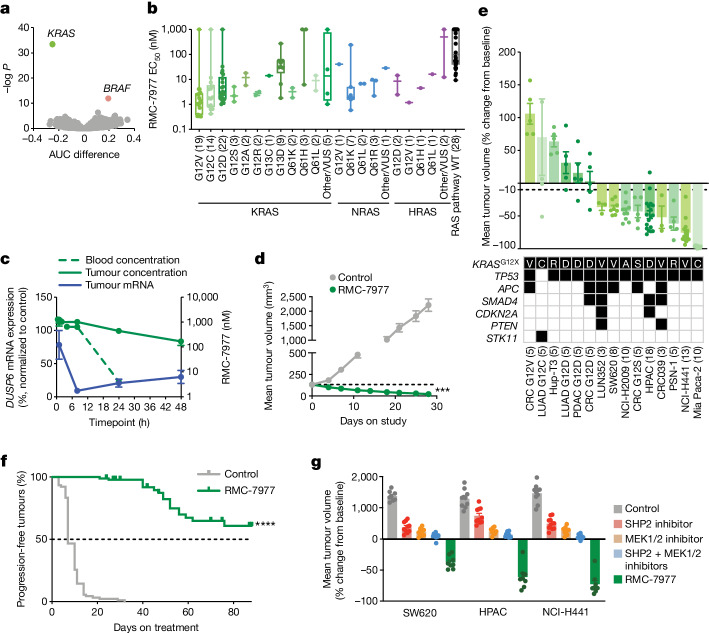
RMC-7977 is broadly active in RAS-addicted cancer models. **a**, Relationship between the area under the curve (AUC) difference (see Supplementary Methods) and negative log-transformed *P* value (two-sided Wilcoxon test) between cell lines by genotype. Points represent mutated genes. Negative AUC indicates sensitivity; positive AUC indicates resistance. **b**, RMC-7977 EC_50_ according to *KRAS* genotype. Each dot represents a cell line. The centre line is the median, box limits represent first and third quartiles and whiskers depict the range. The number of cell lines in each group is indicated in parentheses. VUS, variants of unknown significance. **c**, Blood and tumour concentrations of RMC-7977 (green) and *DUSP6* mRNA (blue) for NCI-H441 xenograft tumours following one oral dose of 10 mg kg^−1^ RMC-7977. Data are mean ± s.e.m. of three biological replicates. **d**, Mice bearing NCI-H441 CDX tumours treated with 10 mg kg^−1^ RMC-7977 orally once daily for 28 days. ***Adjusted *P* value = 0.0002; two-way ANOVA (*n* = 8 mice per group) with multiple comparison Dunnett’s test. The dashed line shows the initial average tumour volume. Data are mean ± s.e.m. for eight mice per group. **e**, KRAS(G12X) xenograft models treated with RMC-7977 (10 mg kg^−1^ by oral administration) for 4–6 weeks. Data are mean ± s.e.m. of 3–18 mice per group. One data point for LUAD G12C is beyond the axis range. Shaded boxes in the table indicate gene variants. **f**, Kaplan–Meier analysis of time to tumour size doubling (*n* = 90 mice per group) of *KRAS*^G12X^ mutant models treated with 10 mg kg^−1^ RMC-7977 orally once daily. **g**, CDX models treated with vehicle control, SHP2 inhibitor (20 mg kg^−1^ RMC-4550 orally every 2 days), MEK inhibitor (2.5 mg kg^−1^ cobimetinib orally once daily), combined SHP2 and MEK inhibitors (20 mg kg^−1^ RMC-4550 orally every 2 days and 2.5 mg kg^−1^ cobimetinib orally once daily), or 10 mg kg^−1^ RMC-7977 orally once daily. NCI-H441 (*KRAS*^G12V^, NSCLC) and HPAC (*KRAS*^G12D^, PDAC) models were treated for 21 days. SW620 (*KRAS*^G12V^, CRC) was treated for 28 days. Data are mean ± s.e.m.; *n* = 8 mice per group for control and RMC-7977, and *n* = 10 mice per group for RMC-4550, cobimetinib, and RMC-4550 + cobimetinib. Source Data

**Extended Data Fig. 7:**
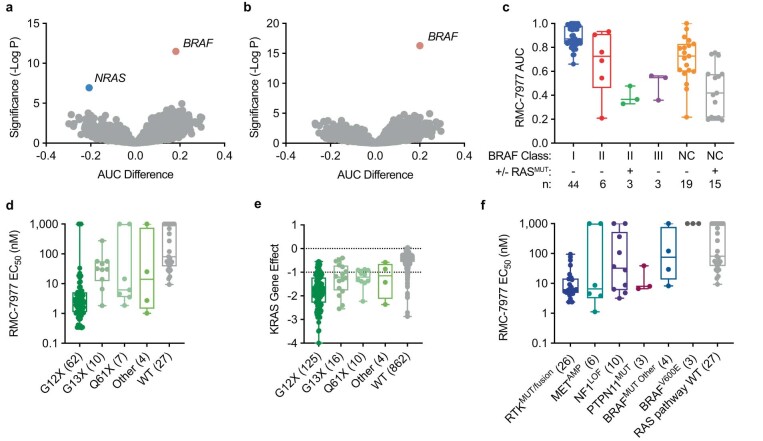
RMC-7977 sensitivity by genotype. **a**,**b**, AUC difference (x-axis) and significance (y-axis, two-sided Wilcoxon) between cell lines by genotype. Each point represents a mutated gene. Negative AUC implies increased sensitivity. *NRAS* correlation shown in (**a**) was only evident when *KRAS* mutants were removed, likely due to the disproportionately larger number of KRAS mutant cell lines. The dataset in (**b**) excluded both *KRAS* and *NRAS* mutants. A small sampling of only 22 *HRAS* mutants with RMC-7977 activity data was insufficient to determine a statistically significant correlation with *HRAS* dependence. **c**, PRISM data highlighting *BRAF* Class I mutations are strongly activating monomers; Class II mutations are moderately activating dimers; Class III are kinase impaired; NC are *BRAF* mutated but have not been classified as I, II, or III; *KRAS*, *NRAS*, or *HRAS* co-mutation status indicated as with + or - for each group (**c**). RMC-7977 EC_50_ (CTG) shown as a function of mutated *KRAS* codon (**d**). *KRAS* gene effect (CHRONOS score from http://depmap.org) plotted as a function *KRAS* genotype. Dotted lines indicate no proliferation effect and inhibition of cell growth (0 and −1 respectively) (**e**). RMC-7977 EC_50_ (CTG) shown as a function of RAS pathway activating mutation. Median EC_50_: RTK^MUT/fusion^ (6.14 nM), *MET*^*AMP*^ (6.61 nM), *NF1*^*LOF*^ (32.5 nM), *PTPN11*^*MUT*^ (7.95 nM), *BRAF*^*MUT Other*^ (75.4 nM), *BRAF*^*V600E*^ ( > 1 µM), RAS pathway wild-type (WT) (71.5 nM). RAS pathway WT represents cell lines without *KRAS, NRAS, HRAS, BRAF* or the aforementioned genetic alterations (**f**). **c**,**d**,**e**,**f**, Points represent cell lines; centre line is the median; box limits depict quartiles; whiskers represent range (n = 3-862 cell lines per genotype). Source Data

We next performed a cell viability assay in 869 human tumour cell lines of different genetic and histological subtypes in a pooled, multiplexed format (PRISM assay) to identify genetic features associated with RMC-7977 sensitivity or resistance. Oncogenic *KRAS* mutation status provided the most significant genetic marker of sensitivity to RMC-7977 (Fig. 3a). Similar results were observed for *NRAS* mutations, although no correlation with *HRAS* mutation status was detected, owing to the low representation of *HRAS* mutations (*HRAS*-mutated *n* = 22; Extended Data Fig. 7a,b). Unsurprisingly, among cell lines with *BRAF* mutations, *BRAF* class I V600 mutations were the most abundantly represented and clearly associated with resistance, as BRAF is a kinase effector of RAS and V600 mutations render BRAF RAS-independent. Cell lines with less common class II or III mutations, which remain somewhat dependent on upstream RAS signalling and frequently co-occur with RAS mutations, were often sensitive to RMC-7977, as were many unclassified *BRAF* mutations (Extended Data Fig. 7c).

We then selected a second, focused panel of 183 individually arrayed human cancer cell lines enriched for RAS and RAS pathway mutations to interrogate RMC-7977 potency. *KRAS*^G12X^ mutant cell lines were highly sensitive to RMC-7977, with a median EC_50_ of 2.40 nM. By comparison, non-G12 mutant KRAS cell lines showed around tenfold reduced sensitivity (median EC_50_ = 25.1 nM) (Fig. 3b), consistent with the *KRAS* gene dependency observed for *KRAS-*mutant cell lines (extended Data Fig. 7d,e). The increased sensitivity observed for *KRAS*^G12X^ mutant cell lines may be owing, in part, to distinct biochemical properties of the various oncogenic *KRAS* mutations^19,20^. Codon 13 and 146 mutations are associated with high nucleotide exchange and are not as highly GTP-bound as codon 12 or 61 mutant RAS proteins^21^. Tissue-specific phenotypes and co-mutation status may also influence RAS dependency^22,23^. Codon 13 mutations are found predominantly in CRCs and are frequently co-mutated with *NF1* or receptor tyrosine kinase (RTK) genes, which may affect RAS dependency and, by extension, RMC-7977 sensitivity^24^. Several *KRAS* wild-type genotypes, including *NRAS* and *HRAS* mutant cell lines (median EC_50_ = 6.76 nM), and cell lines with mutationally activated RTKs also responded to RMC-7977 inhibition, including those with mutations or fusions of *EGFR*, *ERBB3*, *FGFR1*, *FGFR2, FGFR3*, *ROS1*, *RET*, *NTRK1* and *ALK* (median EC_50_ = 6.14 nM), and cell lines with wild-type *MET* gene amplification (median EC_50_ = 6.61 nM; Extended Data Fig. 7f). Cell lines with *NF1* loss of function and *PTPN11* mutations, which each cause activation of wild-type RAS signalling, were moderately sensitive (median EC_50_ = 28.1 nM). Together, these data are concordant with our genetic analysis of *RAS* dependence and support the on-target pharmacological activity of RMC-7977.

**Extended Data Fig. 8:**
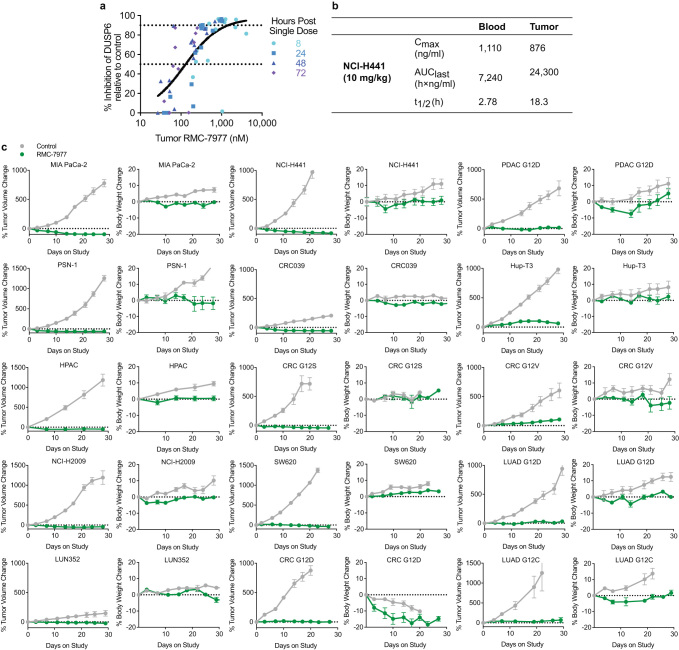
RMC-7977 PKPD, tumour volumes, and body weights of tumour bearing mice. **a**, Pharmacokinetic (PK) relationship to pharmacodynamic (PD) marker (*DUSP6*) levels. RMC-7977 tumour concentrations and percentage of *DUSP6* inhibition in tumour following single oral administration in NCI-H441 (KRAS^G12V^, NSCLC) tumour bearing BALB/c nude mice. All datapoints shown, n = 3 per dose and timepoint. EC_50_ = 130 nM and an EC_90_ = 1,450 nM depicted by horizontal dotted lines, with a maximal level of inhibition near 100% relative to control. **b**, Table of blood and tumour PK parameters of RMC-7977 following single oral administration of RMC-7977 in NCI-H441 tumour bearing BALB/c nude mice. Whole blood and tumour concentrations of RMC-7977 were determined using liquid chromatography-tandem mass spectrometry (LC-MS/MS). PK parameters were calculated by non-compartmental analysis (Phoenix WinNonlin). **c**, Percent tumour volume change and percent body weight change of tumour bearing BALB/c nude mice following repeated oral administration of either vehicle control or RMC-7977 at 10 mg/kg. Treatments were administered daily for 7 days per week except for CRC G12V, LUAD G12D, CRC G12S, LUAD G12C, and PDAC G12D, which were all treated once daily for 5 days of treatment followed by 2 days of treatment cessation every week. Tolerability defined as body weight loss <20%. n = 3–28 mice per group, datapoints represent mean ±s.e.m. normalized to initial (day 0, depicted as a horizontal dotted line) measurements. Source Data

We then assessed the pharmacodynamic and anti-tumour activity of RMC-7977 in vivo in the NCI-H441 CDX model of NSCLC (*KRAS*^G12V^, NSCLC). The relationship between the total tumour concentration of RMC-7977 and inhibition of the RAS pathway transcriptional target *DUSP6* in tumour lysates yielded an EC_50_ of 130 nM (Extended Data Fig. 8a), consistent with the measured KRAS(G12V) *K*_d2_ of 85 nM (Extended Data Table 1), and with the model for tri-complex RAS inhibition discussed above. A single oral dose of 10 mg kg^−1^ RMC-7977 was sufficient to maximally suppress tumour *DUSP6* levels (91%) at 8 h, which partially recovered over 48 h, concordant with the decrease in tumour RMC-7977 concentrations (Fig. 3c). Prolonged RMC-7977 exposure in tumours was observed in this and other subcutaneously implanted xenograft tumour models, resulting in an approximately threefold increase in overall exposure in subcutaneous tumours compared with blood (Extended Data Fig. 8b). Repeated daily administration of RMC-7977 at 10 mg kg^−1^ was well tolerated and resulted in 83% mean tumour regression following 28 days of treatment in the NCI-H441 model (Fig. 3d).

RMC-7977 caused tumour growth inhibition and induced multiple tumour regressions across a larger panel of 15 PDAC, CRC and NSCLC CDX and patient-derived xenograft (PDX) models bearing *KRAS*^G12X^ mutations and co-mutations representative of the genomic landscape of patients with *KRAS*-mutant cancers (Fig. 3e). RMC-7977 treatment resulted in mean tumour regression in 9 out of 15 (60%) models after a 4- to 6-week treatment period (Fig. 3e) and had a minimal effect on body weights in all models (Extended Data Fig. 8c). Of note, when we continued RMC-7977 treatment in these xenograft models for up to 90 days, the anti-tumour activity of RMC-7977 was found to remain durable as the majority of regressions and even cytostatic responses were maintained. Whereas the controls exhibited a short median time to tumour doubling of 7 days, RMC-7977 treated tumours did not reach a median time to tumour doubling (defined as tumour progression) on treatment in a Kaplan–Meier analysis of these results (Fig. 3f; Cox proportional hazard ratio 0.004, 95% interval 0.0011–0.0191, *P* < 1 × 10^−12^).

MEK and ERK inhibitors have undergone extensive clinical testing as monotherapies or in combinations with other RAS pathway inhibitors in patients with *KRAS* or *NRAS* mutated cancers^25^. Despite encouraging preclinical results, these therapeutic strategies have so far been unsuccessful in the clinic^26–28^, with therapeutic benefits probably compromised by dose-limiting toxicities^29–31^. We compared the anti-tumour activity of single agent RMC-7977 to that of the upstream and downstream RAS-MAPK pathway inhibitors RMC-4550 (SHP2 inhibitor) and cobimetinib (MEK inhibitor), respectively, administered as single agents or in combination, in three *KRAS*^G12X^ models. At well-tolerated doses, RMC-7977 induced deep regressions in all three models. By contrast, following administration of MEK and SHP2 inhibitors at doses that were well-tolerated and translatable, either alone or in combination, only modest tumour growth inhibition was observed (Fig. 3g). These data demonstrate that in these preclinical models of *KRAS*^G12X^ mutant cancers, direct targeting of active RAS with RMC-7977 elicits a differentiated and superior anti-tumour activity profile compared with upstream and/or downstream vertical inhibition of the oncogenic driver.

There are several potential explanations for why RMC-7977 elicits greater anti-tumour activity in *KRAS*^G12X^-driven cancers compared with agents that target upstream or downstream nodes on the RAS pathway. These include more efficient suppression of oncogenic RAS signalling, relatively less impact on normal tissues^32^, or a combination of both. Directly targeting the RAS oncoprotein itself may exploit the high degree of oncogene addiction of *KRAS*^G12X^ (and *NRAS*)-mutated cancer cells to a greater degree than targeting upstream and downstream signalling proteins (such as SHP2, MEK1/2 and ERK1/2). Furthermore, whereas MEK inhibition did not distinguish between wild-type and mutant RAS variants (Extended Data Fig. 6a), RMC-7977 exhibited modestly lower potency and incomplete wild-type RAS suppression compared with KRAS(G12X) in cells (Fig. 2b,e and Extended Data Table 2). Additionally, the slow elimination of RMC-7977 observed in subcutaneous xenograft tumours relative to blood (Fig. 3c and Extended Data Fig. 8b) suggests that it is differentially distributed to tumours, which may contribute to a wider therapeutic index. Of note, *PPIA* mRNA expression is reportedly induced by hypoxia under control of *HIF1A* and has a critical role in tumorigenesis^33,34^. Consistent with these reports, subcutaneous xenograft tumours express increased amounts of CYPA protein compared with cells grown in vitro under normoxic conditions (Extended Data Fig. 5d) and *PPIA* mRNA expression is increased in tumour cells^35^. Collectively, these data support the notion that CYPA is critical for tumour maintenance and may also affect tumour distribution and cellular retention of tri-complex inhibitors.

**Extended Data Fig. 9:**
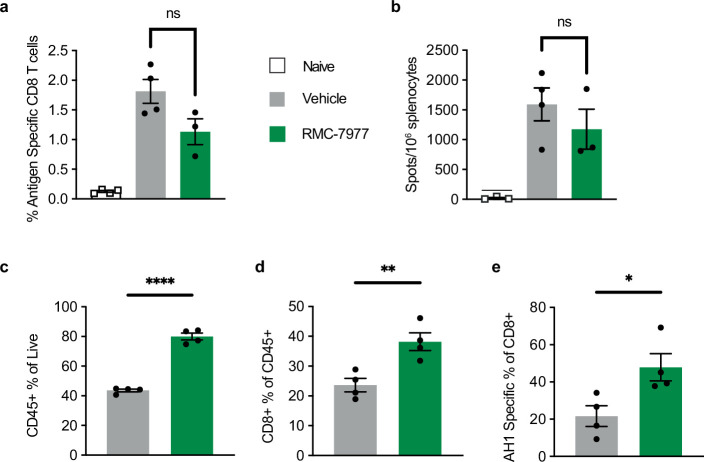
T cell response in both naïve and tumour-bearing immunocompetent mice treated with RMC-7977. **a**,**b**, C57BL/6 J mice were vaccinated with 1×10^6^ OVA peptide (SIINFEKL)-pulsed BMDCs on day 0 and day 8. Mice were treated with 25 mg/kg RMC-7977 or vehicle PO daily starting one day before vaccination (day −1). Mice were euthanized on day 15 after treatment start, and spleens were harvested and assessed for frequency of antigen specific (H-2Kb SIINFEKL tetramer positive) CD8 + T cells by flow cytometry (**a**) and for IFNγ production by ELISpot. Quantification shown in (**b**). Each bar represents mean ±s.e.m; each dot represents an individual mouse. n = 3-4 mice/group; ns=not significant (unpaired two-sided Student’s t test). **c**,**d**,**e**, Levels of immune cells, CD8 + T cells, and tumour antigen (AH1) specific CD8 + T cells in murine colon carcinoma CT26 syngeneic tumours, engineered to express KRAS^G12C^, at 24 h post 4 days of treatment with RMC-7977 at 25 mg/kg PO QD shown as percentage of Live (**c**), CD45+ (**d**), and CD8+ cells (**e**), n = 4 mice per group, bars represent mean ± s.e.m, * p = 0.0286; ** p = 0.0079; **** p = 0.000006 by unpaired two-sided Student’s t test. Source Data

We interrogated the potential for RMC-7977-mediated inhibition of wild-type RAS to impair immune cell function in both naive and tumour-bearing immunocompetent mice. Tumour-naive mice were able to mount a CD8^+^ T cell response to ovalbumin peptide vaccination in the presence of RMC-7977 treatment (Extended Data Fig. 9a,b). Furthermore, RMC-7977 increased tumour-antigen-specific CD8^+^ T cell infiltration into *KRAS*^G12C^ syngeneic tumours (Extended Data Fig. 9c–e).

## Overcoming KRAS(G12C) OFF resistance

**Fig. 4:**
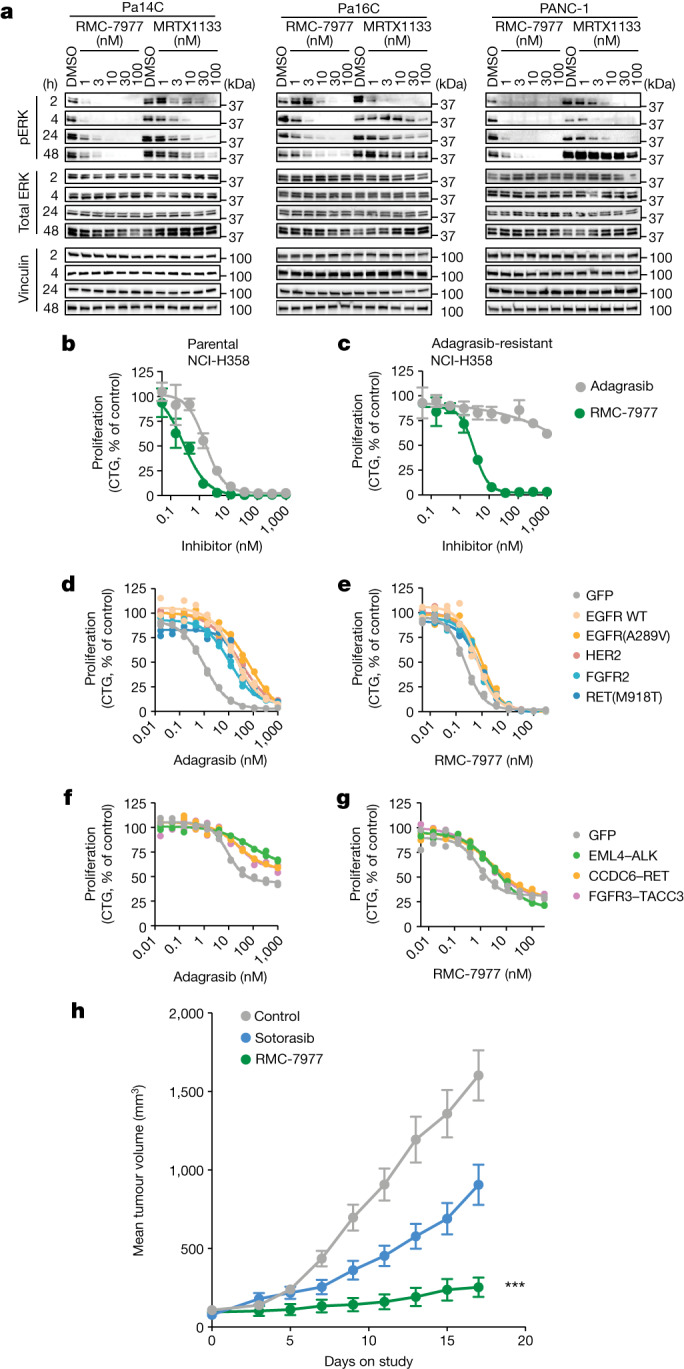
RMC-7977 can overcome resistance to mutant-selective KRAS inhibition. **a**, Western blots showing the time course of RAS signalling in *KRAS*^G12D^ PDAC cell lines treated with RMC-7977, MRTX1133 or DMSO control. Total ERK and vinculin were used as loading controls. Data are representative of two similar experiments. **b**,**c**, Parental NCI-H358 cells (*KRAS*^G12C^, NSCLC) (**b**) and adagrasib-resistant NCI-H358 cells with a secondary *NRAS*^Q61K^ mutation (**c**) were treated with adagrasib or RMC-7977 for 5 days and proliferation was measured by CTG assay. **d**,**e**, NCI-H358 (*KRAS*^G12C^, NSCLC) cells expressing exogenous RTK DNA constructs as indicated (GFP control, wild-type EGFR, EGFR(A289V), HER2, FGFR2 or RET(M918T)) were treated with adagrasib (**d**) or RMC-7977 (**e**) for 120 h, and proliferation was measured by CTG assay. **f**,**g**, MIA PaCa-2 (*KRAS*^G12C^, PDAC) cells expressing exogenous RTK fusion DNA constructs as indicated (GFP control, EML4–ALK, CCDC6–RET or FGFR3–TACC3) were treated with adagrasib (**f**) or RMC-7977 (**g**) for 120 h, and proliferation was measured by CTG assay. **d**–**g**, Biological duplicates normalized to vehicle control are shown from one of 2–5 independent experiments. **h**, Patient-derived xenograft model established from a patient with *KRAS*^G12C^ NSCLC who developed resistance after sotorasib. Mice were treated with vehicle (*n* = 7), sotorasib (50 mg kg^−1^ orally once daily; *n* = 7), or RMC-7977 (10 mg kg^−1^ orally once daily; *n* = 10). Tumour volumes were assessed for 17 days after treatment started. ***Adjusted *P* value = 0.0001 for RMC-7977 versus control group; repeated measures two-way ANOVA adjusted based on multiple comparison via Dunnett’s test on the final tumour measurement. Data are mean ± s.e.m. *n* refers to the number of mice in each group. Source Data

Although inactive-state KRAS(G12C) inhibitors provide short-term therapeutic benefit for some patients, most eventually relapse through acquired genetic or adaptive mechanisms of resistance^36–39^. Ryan et al.^39^ reported that adaptive feedback reactivation of upstream RTK signalling through wild-type RAS limits the activity of KRAS(G12C) inhibitors. We observed analogous results in KRAS(G12D) mutant PDAC cell lines treated with the KRAS(G12D)-selective inhibitor, MRTX1133, in which pERK suppression seen at 2 h rebounded by 48 h after treatment (Fig. 4a). We hypothesized that RMC-7977 could address adaptive RAS signalling mechanisms that rely on increased active-state wild-type and mutant RAS proteins. Consistent with this hypothesis, RMC-7977 showed sustained pERK suppression in *KRAS*^G12D^ PDAC cells in culture for 48 h, suggesting that broad inhibition of RAS family proteins can overcome the adaptive feedback observed with mutant-selective inhibitor treatment (Fig. 4a). Similar sustained pERK suppression and moderate PARP cleavage were also observed in two additional *KRAS*-mutant cancer cells (Extended Data Fig. 6b,c). We therefore hypothesized that the concurrent suppression of wild-type and mutant RAS signalling could drive durable anti-tumour responses to RMC-7977 treatment in vivo. As described above, a 90-day treatment study in a series of KRAS(G12X) xenograft models demonstrated a marked and significant increase in time to tumour doubling from baseline compared with controls (Fig. 3f).

**Extended Data Fig. 10:**
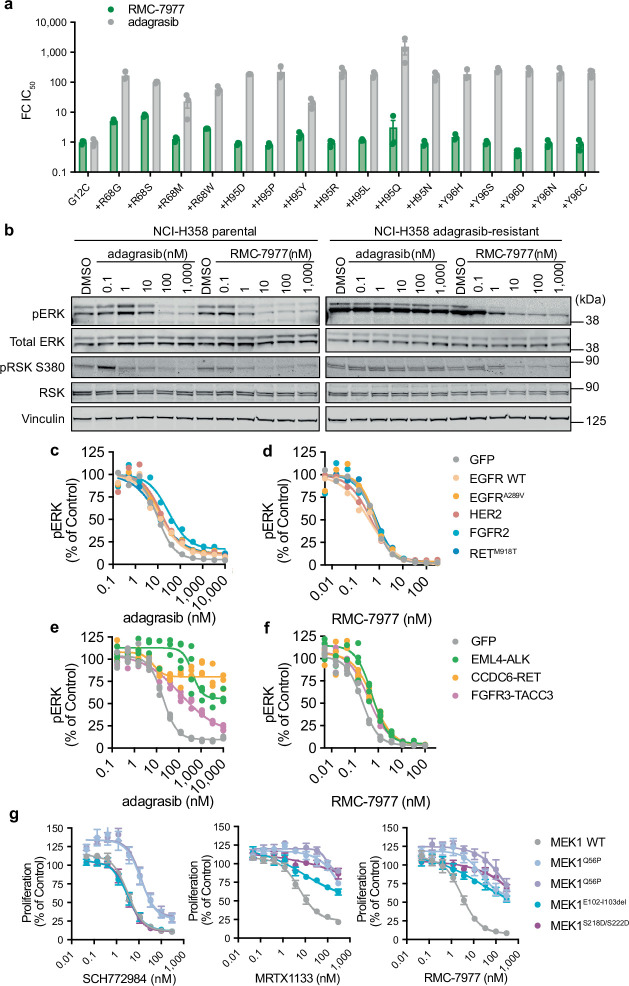
RAS^MULTI^ inhibition overcomes G12C inhibitor resistance mechanisms. **a**, Cellular nano-BRET assay showing fold-change IC_50_ of disrupting the KRAS:CRAF interaction in U2O2 cells expressing KRAS^G12C^ alone or with the indicated secondary mutation in the SWII domain and treated with RMC-7977 or adagrasib for 4 h. Bars represent mean of n = 3 biological replicates ±s.e.m. **b**, Western blots of parental NCI-H358 (KRAS^G12C^, NSCLC) and an adagrasib resistant clone of NCI-H358 cells with a secondary NRAS^Q61K^ mutation. Cells were treated with adagrasib or RMC-7977 for 4 h. **c**,**d**, pERK levels (MSD) in NCI-H358 (KRAS^G12C^, NSCLC) cells expressing exogenous RTK DNA constructs indicated by color (GFP control, EGFR^WT^ EGFR^A289V^, HER2, FGFR2, or RET^M918T^, treated with adagrasib (**c**) or RMC-7977 (**d**) for 24 h. **e**,**f**, pERK levels (MSD) in MIA PaCa-2 (KRAS^G12C^, PDAC) cells expressing exogenous RTK fusion DNA constructs indicated by color (GFP control, EML4-ALK, CDC6-RET, FGFR3-TACC3), treated with adagrasib (**e**), or RMC-7977 (**f**) for 24 h. n = 2–4 biological replicates per group, normalized to control. Data shown are representative of independent experiments (NCI-H358 n = 3, MIA PaCa-2: n = 2). **g**, Pa16C (KRAS^G12D^, PDAC) cells expressing exogenous MEK1 mutant DNA constructs were treated with the indicated inhibitors for 120 h, and proliferation was measured by Calcein AM. n = 3–5 biological replicates from a single experiment, datapoints represent mean ± s.e.m normalized to control. Source Data

The activity of RMC-7977 against multiple forms of oncogenic RAS suggests the potential for therapeutic benefit against resistance mechanisms involving secondary RAS mutations. Tri-complex KRAS(G12C) (ON) inhibitors, such as RMC-4998, bind to RAS through a unique mechanism and a binding site distinct from the switch II pocket occupied by inactive-state KRAS(G12C) inhibitors, such as adagrasib and sotorasib^12,40–42^. Switch II pocket binding mutations such as those at positions R68, Y96 and H95 had little or no effect on RMC-7977 potency (Extended Data Fig. 10a), as was observed for RMC-4998 (ref. ^36^). Next, we tested whether the broad-spectrum RAS inhibitory activity of RMC-7977 could counter additional genetic resistance mechanisms observed in relapsed patients treated with KRAS(G12C) inhibitors, including secondary oncogenic RAS mutations and RTK activation. Indeed, RMC-7977 inhibited RAS signalling and growth of a NCI-H358 (*KRAS*^G12C^, NSCLC) clone with a concurrent *NRAS*^Q61K^ mutation that emerged in cells grown under continuous exposure to adagrasib in vitro (Fig. 4b,c and Extended Data Fig. 10b). RTK amplification and activating mutations can also cause RAS pathway reactivation through mutant and wild-type RAS proteins. We used an engineered system with doxycycline-inducible constructs of full-length and fusion RTKs previously detected in patients who progressed on adagrasib or sotorasib treatment^36^. Overexpression of wild-type or mutant RTKs in NCI-H358 cells (*KRAS*^G12C^, NSCLC) conferred reduced sensitivity to adagrasib (proliferation inhibition EC_50_ shift: wild-type *EGFR*, 42-fold; *EGFR*^A289^, 153-fold; *HER*, 51-fold; *FGFR*, 18-fold, *RET*^M918^, 34-fold), but not to RMC-7977 (proliferation inhibition EC_50_ shift ≤ 3-fold) (Fig. 4d,e). A similar trend was seen for inhibition of pERK inhibition (Extended Data Fig. 10c,d). Similar results were observed when oncogenic RTK fusion proteins (EML4–ALK, FGFR3–TACC3 and CCDC6–RET) were exogenously expressed in MIA PaCa-2 cells (*KRAS*^G12C^, PDAC) (Fig. 4f,g and Extended Data Fig. 10e,f). As expected, downstream MEK1 mutations conferred resistance to both OFF state and ON state RAS inhibitors (Extended Data Fig. 10g).

Finally, we examined RMC-7977 treatment in a KRAS(G12C)-mutated PDX model derived from a NSCLC patient who had achieved stable disease on sotorasib but quickly relapsed. Genomic alterations in this tumour include amplification of the wild-type *KRAS* allele accompanied by increased levels of GTP-KRAS (M. Nokin et al., unpublished observations), which contributes to diminished response to sotorasib treatment at 50 mg kg^−1^ daily. RMC-7977 administered daily at 10 mg kg^−1^ resulted in significant anti-tumour activity, with 90% inhibition of tumour growth observed at day 17 of treatment, whereas sotorasib treatment induced only 47% tumour growth inhibition (Fig. 4h). In sum, these data indicate that both adaptive and acquired mechanisms of resistance to KRAS(G12C) inhibitors that lead to RAS pathway reactivation are susceptible to inhibition by RMC-7977.

RMC-7977 extends the tri-complex inhibitor strategy to non-covalently target the active state of wild-type and multiple oncogenic RAS variant proteins, with particular activity against the range of common codon 12 mutants, thus offering therapeutic potential for RAS(ON) multi-selective inhibitor across a spectrum of RAS-addicted cancers, including NSCLC, CRC and PDAC. Evidence of robust, durable anti-tumour activity at well-tolerated doses across various RAS mutant xenograft models provides preclinical validation for the direct targeting of active RAS variants as a desirable therapeutic strategy. Furthermore, we demonstrate that concurrent inhibition of multiple oncogenic RAS variants and wild-type RAS in the same tumour cell with a reversible broad-spectrum RAS^MULTI^ inhibitor such as RMC-7977 can overcome some of the resistance mechanisms recognized to limit the efficacy and durability for inactive-state KRAS(G12C) inhibitors. The proximity of the RMC-7977 binding site to RAS mutational hotspots (residues G12, G13 and Q61) presents a unique opportunity to expand this approach further by designing additional mutant-selective tri-complex inhibitors. Moreover, RAS(ON) multi-selective inhibitors could also provide therapeutic benefit in combination with mutant-selective KRAS inhibitors to improve anti-tumour response by blocking adaptive pathway reactivation and preventing escape through emergence of secondary oncogenic RAS or RTK mutations. The investigational agent RMC-6236 is a first-in-class broad-spectrum RAS(ON) multi-selective protein inhibitor that is currently undergoing clinical evaluation (ClinicalTrials.gov identifier: NCT05379985).

## Methods

### Cell culture and reagents

Most cell lines were obtained from ATCC (listed in Supplementary Methods). Pa14C and Pa16C cells were a gift from A. Maitra, and the MEF cell lines were obtained from the NIH. AsPC-1 *CYPA*-knockout (KO), NCI-H441 CYPA-KO and eCT26 *KRAS*^G12C/G12C^ *ABCB1*^−/−^ cells were generated by Synthego (eCT26 *KRAS*^G12C/G12C^ *ABCB1*^−/−^ was engineered from the mouse CT26 *KRAS*^G12D/G12D^ tumour cell line; P-glycoprotein (PGP) drug transporter was knocked out to eliminate any confounds due to potential interaction of the test article with PGP). All cells were grown in recommended medium supplemented with 10% fetal bovine serum (FBS) and 1% penicillin/streptomycin and maintained at 37 °C in a humidified incubator at 5% CO_2_. The sanglifehrin A-competitive CYPA inhibitor^17^ was synthesized at WuXi AppTec. Other tool inhibitors were acquired from Selleckchem or MedChemExpress.

### Protein production

His_6_-TEV-KRAS4B^WT^ (residues 1–169), His_6_-TEV-KRAS4B^G12A^ (residues 1–169), His_6_-TEV-KRAS4B^G12C^ (residues 1–169), His_6_-TEV-KRAS4B^G12D^ (residues 1–169), His_6_-TEV-KRAS4B^G12R^ (residues 1–169), His_6_-TEV-KRAS4B^G12S^ (residues 1–169), His_6_-TEV-KRAS4B^G12V^ (residues 1–169), His_6_-TEV-HRAS^WT^ (residues 1–166), His_6_-TEV-NRAS^WT^ (residues 1–172), His_6_-TEV-AviTag-KRAS4B^G12C^ (residues 1–169), His_6_-TEV-CYPA (full-length), His_6_-TEV-AviTag-CYPA (full-length) and GST-TEV-BRAF (residues 155–229) were expressed from a pET28 vector in BL21(DE3) *Escherichia coli* and purified as described^12^.

### Crystallography

Conditions, data collection, and refinement protocols are provided in the Supplementary Methods.

### RAS–RAF and RAS–CYPA TR-FRET

Time-resolved fluorescence resonance energy transfer (TR-FRET) was used as previously described to assess disruption of the interactions between wild-type RAS or the mutant oncogenic RAS proteins and the RAS-binding domain of BRAF, and to assess the induction of interactions between the RAS proteins and CYPA^12^.

### CYPA binding affinity

The binding affinity of compounds for CYPA (*K*_d1_) was assessed by SPR on a Biacore 8K instrument as previously described^12^.

### RAS binding affinity

The binding affinity of compound-bound CYPA for the mutant oncogenic RAS proteins (*K*_d2_) mentioned was assessed by SPR on a Biacore 8 K instrument. AviTag-RAS [residues 1–169] was immobilized on a streptavidin sensor chip, and varying compound concentrations were flowed over the chip in assay buffer (10 mM HEPES-NaOH pH 7.4, 150 mM NaCl, 0.005% v/v surfactant P20, 2% v/v DMSO, 25 μM CYPA). The SPR sensorgrams were fit using either a steady state affinity model or a 1:1 binding (kinetic) model to assess the dissociation constant (*K*_d_) for RAS binding.

### AlphaLISA and MSD analysis of cellular ERK phosphorylation

Cells were seeded in 384- or 96-well tissue culture-treated plates in 2D and incubated overnight before exposure to serial dilutions of compound or DMSO control (0.1% v/v). Cells were lysed and the levels of ERK phosphorylation determined using the AlphaLISA SureFire Ultra pERK1/2 (T202/Y204) Assay kit (Perkin Elmer ALSU-PERK-A50K) or MesoScale Discovery (MSD) Multi-Array Assay Systems for Phospho/Total ERK1/2 Whole Cell Lysate Kit (K15107D) according to manufacturers’ protocols. Signal was detected using a Perkin Elmer Envision with standard AlphaLISA settings or a Meso QuickPlex SQ120 reader. For AlphaLISA, raw signal was normalized to vehicle control and a low-signal control compound ((sample signal – average signal of low control)/(average signal for vehicle – average signal for low control) × 100%). The MSD signal of pERK1/2 was divided by the MSD signal for total ERK1/2. The ratio was normalized to vehicle pERK/total ERK (%) = ((pERK/total ERK in treatment condition)/(ratio pERK/total ERK in DMSO control)) × 100%. Data were plotted as a function of log [compound (M)] with a sigmoidal concentration response (variable slope) model fitted to the data to estimate the inhibitor EC_50_ in Prism 9 (GraphPad).

### Cell proliferation analysis

Cells were seeded in 384- or 96-well tissue culture-treated plates in 2D and incubated overnight. Alternatively, cells were seeded in round-bottom ultra-low attachment 96-well plates, centrifuged at 1,000 rpm for 10 min to pellet the cells, and incubated overnight or up to 72 h to allow for 3D spheroid formation. Cells were exposed to serial dilutions of compound or DMSO control (0.1% v/v) for 120 h. Cell viability was determined by CellTiter-Glo 2.0 reagent (2D CTG) (Promega, G9243) or 3D CellTiter-Glo reagent (3D CTG) (Promega, G9683) according to the manufacturer’s protocols. Luminescence was detected using a SpectraMax M5 Plate Reader (Molecular Devices) of Perkin Elmer Enspire. Luminescence signal was normalized to vehicle-treated wells (normalized signal (%) = (luminescence (treated)/mean luminescence (vehicle)) × 100%). For PSN1 and HUPT3, raw signal was normalized to vehicle control and a low-signal control compound ((sample signal – average low control signal)/(average vehicle signal – average low control signal) × 100%). For NCI-H441 and AsPC-1 cells treated with the combination of RMC-7977 and the sanglifehrin A competitive CYPA inhibitor (3 mM), luminescence signal was normalized to that of the CYPA inhibitor treatment-only control (normalized signal (%) = (luminescence (treated)/mean luminescence (CYPA inhibitor only) × 100%)). Data were plotted as log [inhibitor (M)], and a four-parameter sigmoidal concentration response model was fitted to the data to calculate EC_50_. Data were fit with top plateau constrained to 100% and lower plateau constrained depending on the cell line (Capan-1, AsPC-1 and Hs 766T, 25% ≥ lower plateau ≥ 0%; HCT 116, SKMEL30 and KU1919, 10% ≥ lower plateau ≥ 0%; NCI-H358, A375 PSN1 and HUPT3, lower plateau = 0%). Pa16C MEK1 mutant cells were evaluated by live-cell counting using Calcein AM and a SpectraMax i3X multi-mode detection platform (Molecular Devices). Growth percentages were calculated by normalizing the treated cell counts to their respective untreated cell counts.

### Cellular RAS–RAF and RAS–CYPA assays

U2OS cells or U2OS cells with *PPIA* gene knockout were seeded at 500,000 cells per well in a 6-well plate and incubated overnight. KRAS4B, or other small GTPases, containing the indicated mutations were cloned in pNLF-N or pHTN plasmids for expression with an N-terminal nanoluciferase or HaloTag fusion, respectively. Full-length CYPA was cloned into pHTN, the RBD of RAF1 (residues 51–149) was cloned into pHTC, full-length RALGDS was cloned into pHTC, PIK3CA was cloned into pNLF-N, and the catalytic domain of SOS1 (residues 558–1049) was cloned into pNLF-N. U2OS cells were transfected with KRAS and effector plasmids, and U2OS *PPIA*-KO cells were transfected with small GTPase and CYPA plasmids, both using Fugene HD reagent according to manufacturer protocols. The following day, the cells were collected by Trypsin and reseeded in a white tissue culture-treated 96-well plate in OptiMem phenol red-free medium (Gibco) containing 4% FBS and a 1:1,000 dilution of NanoBRET 618 HaloTag ligand (Promega). For endpoint concentration response curves, vivazine nanoluciferase substrate was added to 1× concentration in OptiMem phenol red-free medium with 4% FBS. Varying concentrations of inhibitor were added and incubated for 1 or 4 h before the nano-BRET signal was measured on a Perkin Elmer Envision plate reader. For kinetic assays, endurazine nanoluciferase substrate was used in place of vivazine, and the plate was placed in a Cytation5 multi-mode reader pre-equilibrated to 37 °C and 5% CO_2_. After 1 h of equilibration, RMC-7977 (50 nM) was added and the nano-BRET signal measured.

### Generation of NCI-H358 expressing low and high CYPA

NCI-H358 cells were transduced by lentivirus encoding Cas9, a guide RNA targeting *PPIA* (which encodes CYPA), and the puromycin resistance gene at WarpDrive Bio. Following puromycin selection, Flag–CYPA was introduced under the control of a tet-inducible promoter by lentivirus. Clones with high and low expression levels of Flag–CYPA were isolated at Revolution Medicines. Flag–CYPA expression was induced by adding doxycycline (0.1 µg ml^−1^) for at least 24 h.

### Generation of cell lines with acquired resistance to adagrasib

NCI-H358 cells resistant to adagrasib were generated by continuously culturing in growth medium containing 1 µM adagrasib for approximately 2 months. Resistant cells were subsequently maintained in culture medium containing 1 µM adagrasib, which was removed during assays.

### Generation of inducible full-length and fusion RTK overexpression cell lines

Plasmids encoding the tet-controlled transcriptional silencer (tTS), reverse tet-controlled transcriptional activator (rtTA), and tet-inducible receptor tyrosine kinases (RTKs) and fusion RTKs were synthesized and packaged into lentivirus at Vector Builder. Lentivirus transductions were performed with addition of polybrene (4 µg ml^−1^). NCI-H358 and MiaPaCa2 cells were transduced with lentivirus encoding tTS or rtTA for 48 h prior to selection with blasticidin (5 µg ml^−1^) for 12 days. The concentration of blasticidin was subsequently lowered to 2 µg ml^−1^. NCI-H358 tTS/rtTA cells were then transduced with lentivirus encoding tet-inducible GFP, EGFR, EGFR(A289V), HER2, FGFR2 and RET(M918T). MiaPaCa2 tTS/rtTA cells were transduced with lentivirus encoding GFP or tet-inducible EML4–ALK, FGFR3–TACC3 and CCDC6–RET. Cells were cultured in growth medium containing puromycin (2 µg  ml^−1^) and blasticidin (2 µg ml^−1^) starting 48 h after transduction to maintain selective pressure for both plasmids. Expression of the transgene was induced by adding doxycycline (0.1–1 µg ml^−1^) for at least 24 h.

### Generation of Pa16C cells expressing MEK1 mutants

MEK1 mutants were generated using quick change mutagenesis in MEK1-GFP (Addgene plasmid #14746). MEK1 was PCR amplified with flanking NheI and AgeI sites and digested. Luciferase-PCW107-V5 (Addgene plasmid #64649) was also digested with NheI and AgeI, removing luciferase, and ligated with the MEK1 insert in front of the V5 tag. Lentivirus was made from the control construct (Luciferase-PCW107-V5) and each of the MEK1 constructs by co-transfection with psPax packaging plasmid into HEK293T cells using Fugene 6 Transfection Reagent (Promega). Viral supernatant was collected, combined with polybrene (8 µg ml^−1^), and used to transduce Pa16C (PDAC, KRAS(G12D)) cells in DMEM supplemented with 10% FBS. Cells were infected for 12 h and then selected using puromycin.

### PRISM assay

RMC-7977 was screened in 931 PRISM DNA-barcoded cell lines established by the Broad Institute. In brief, 20–25 cell lines per pool were plated in 384 well plates and treated with RMC-7977 at 8 doses in threefold dilutions starting at 10 µM for 5 days. Cells were then lysed in TCL mRNA lysis buffer, and then PCR with reverse transcription was performed. Detection of the barcodes and univariate and multivariate analysis was then performed as previously described^43^. Data analysis is described in the Supplementary Methods. Up-to-date code for our analysis is at the github link: https://github.com/cmap/dockerized_mts.

### Cell panel

A panel of 183 cancer cell lines harbouring mutant and wild-type RAS was screened for response to RMC-7977 by cell proliferation and viability inhibition at Crown Bioscience. The panel consisted of cell lines with any substitution at position 12 of KRAS, NRAS or HRAS (KRAS(G12X), NRAS(G12X), HRAS(G12X)); substitutions in KRAS, NRAS or HRAS at any position other than 12, 13 and 61 (KRAS(other/VUS), NRAS(other/VUS), HRAS(other/VUS)); other oncogenic mutations in the RAS pathway (ABL1, ALK, ARAF, BRAF, CBL, EGFR, ERBB2, ERBB3, ERBB4, ERRFI1, FGFR1, FGFR2, FGFR3, FGFR4, FLT3, HRAS, IGF1R, JAK2, KIT, MAP2K1, MAP2K2, MAPK1, MET, NF1, NRAS, NTRK1, NTRK2, NTRK3, PDGFRA, PTPN11, RAC1, RAF1, RASA1, RET, RIT1, ROS1 and SOS1); and no oncogenic mutations in the RAS pathway. Cells were cultured in methylcellulose and treated in triplicate with serial dilutions of RMC-7977 or DMSO. Cells were incubated for 120 h, and cell viability was determined using the CellTiter-Glo Luminescent Cell Viability Assay (CTG) (Promega, G7572) according to the manufacturer’s instructions. Data were plotted as a function of log [inhibitor (M)] and a four-parameter sigmoidal concentration response model was fitted to the data to estimate the inhibitor EC_50_ using Genedata Screener.

### Western blot analysis

Antibodies and protocols are described in the Supplementary Methods.

### Quantification of CYPA protein level in cell and tumour samples

Cells were seeded at 1 × 10^6^ cells per well in triplicate in 6-well plates and incubated overnight. The following day, cells were collected by Trypsin, washed in PBS, pelleted by centrifugation, and snap frozen in a slurry of dry ice and ethanol. Tumour samples were collected and flash frozen (see Supplementary Methods). Samples were transferred to IQ Proteomics for analysis. The samples were lysed by bead beating in 8 M urea + 200 mM EPPS pH 8.0 + HALT protease inhibitor cocktail. Following bead beating, SDS was added to the lysate, 1% final (w/v). Following quantification by BCA assay, lysate corresponding to 16 μg of total protein was aliquoted for downstream processing. Samples were reduced and alkylated via DTT/Iodoacetamide, and protein was isolated via ethanol precipitation. Protein was digested in 100 mM EPPS pH 8.1, using LysC (overnight, room temperature) and Trypsin (6 h, 37 °C). 5 stable isotope labelled standard peptides spanning the CYPA protein sequence (sequences VSFELFADK, ALSTGEK, FEDENFILK, TEWLDGK and EGMNIVEAMER) were spiked into each sample at a ratio of 25 fmol μg^−1^ total protein digested. Endogenous (light) and internal standard (heavy) peptides were quantified via custom targeted assay on an Orbitrap Lumos instrument (Thermo).

### Bioanalysis of cells and supernatant

Ten-million cells were exposed to RMC-7977 (10, 100 or 1,000 nM) in suspension at 1 × 10^6^ cells ml^−1^ for 1 h at 37 °C. Cells were pelleted by centrifugation, and 1 ml of supernatant was reserved and frozen at −80 °C. Cell pellets were washed twice in cold PBS, and pre-weighed tubes containing the cell pellets were weighed prior to snap freezing in a slurry of dry ice and ethanol. Concentrations of RMC-7977 in cell pellets and supernatant were determined by liquid chromatography–tandem mass spectrometry (LC–MS/MS) methods. Cell pellet samples were resuspended in cell medium (diluted as needed), then treated as supernatant. An aliquot of supernatant or resuspended cells (50 µL) was quenched with a 3× volume of acetonitrile containing the internal standard terfenadine (2.5 ng ml^−1^). Samples were vortexed, centrifuged, and analysed on a Sciex 6500+ triple quadrupole mass spectrometer equipped with a Shimadzu AD LC system. A Waters ACQUITY UPLC BEH C4 1.7 µm (2.1 × 50 mm) column was used with gradient elution for compound separation. RMC-7977 and internal standard were detected by positive electrospray ionization using multiple reaction monitoring (RMC-7977: *m*/*z* 865.273/833.500; terfenadine: *m*/*z* 471.939/436.300). The lower limit of quantification was 0.25 ng ml^−1^, and the calibration range was 0.25 to 400 ng ml^−1^. The intracellular concentration of RMC-7977 was calculated using the mass of each cell pellet (mass of empty tube subtracted) and the known cell number, with the assumptions that the volume of a cell is ~2,000 µm^3^, that the density of a cell is approximately the density of water (thus, cell volume = cell mass); and that any compound in CYPA-KO cells in excess of the medium concentration is probably membrane-bound. The ratio of compound concentration in the cell pellet to compound in medium was determined for each concentration of RMC-7977 tested.

### Animal studies

Xenograft studies were conducted at GenenDesign, Pharmaron, Wuxi AppTec, the laboratory of P. Lito, and the laboratory of C. Ambrogio. Animals were assigned to study groups using stratified randomization based upon their tumour volumes. All procedures related to animal handling, care and treatment were conducted in compliance with all applicable regulations and guidelines of the relevant Institutional Animal Care and Use Committee (IACUC). For the sotorasib-resistance xenograft study, all procedures and animal housing conformed to the regulatory standards and were approved by the Italian Health Minister (authorization no. 1227/2020-PR); all experiments were performed in accordance with the guideline for Ethical Conduct in the Care and Use of Animals as stated in The International Guiding Principles for Biomedical Research Involving Animals, developed by the Council for International Organizations of Medical Sciences. Experimental details are supplied in the Supplementary Methods.

### Mouse blood and tumour sample bioanalysis

Whole-blood and tumour concentrations of RMC-7977 were determined using LC–MS/MS methods performed at WuXi AppTec. Tumour tissue samples were homogenized with a 10× volume of methanol/15 mM PBS (1:2, v:v). Sample preparation and analysis on a Sciex 6500+ triple quadrupole mass spectrometer equipped with an ACQUITY UPLC system were performed as previously described^12^. RMC-7977 and internal standard verapamil were detected by positive electrospray ionization using multiple reaction monitoring (RMC-7977: *m*/*z* 865.4/706.4; verapamil: *m*/*z* 455.2/164.9).

### In vivo pharmacodynamic analysis by *DUSP6* qPCR

RNA extraction and analysis of *DUSP6* levels by in tumour tissue were performed as previously described^12^.

### OVA peptide vaccination

Experimental details are described in Supplementary Methods.

### Immune cell response in vivo

Experimental details are described in Supplementary Methods.

### Ethics statement

All CDX and PDX mouse efficacy and pharmacodynamics and pharmacokinetics studies and procedures related to animal handling, care and treatment were conducted in compliance with all applicable regulations and guidelines of the relevant Institutional Animal Care and Use Committee (IACUC). For the sotorasib-resistance PDX studies, all experiments were performed in accordance with the guideline for Ethical Conduct in the Care and Use of Animals as stated in The International Guiding Principles for Biomedical Research Involving Animals, developed by the Council for International Organizations of Medical Sciences.

### Reporting summary

Further information on research design is available in the Nature Portfolio Reporting Summary linked to this article.

## Online content

Any methods, additional references, Nature Portfolio reporting summaries, source data, extended data, supplementary information, acknowledgements, peer review information; details of author contributions and competing interests; and statements of data and code availability are available at 10.1038/s41586-024-07205-6.

### Supplementary information

Supplementary Methods

Reporting Summary

Supplementary Table 1 Table of data collection and refinement statistics (molecular replacement) for crystal structures.

Unprocessed western blots

### Source data

Source Data Figs. 1–4 and Source Data Extended Data Figs. 1–10

## Figures and Tables

**Extended Data Table 1:**
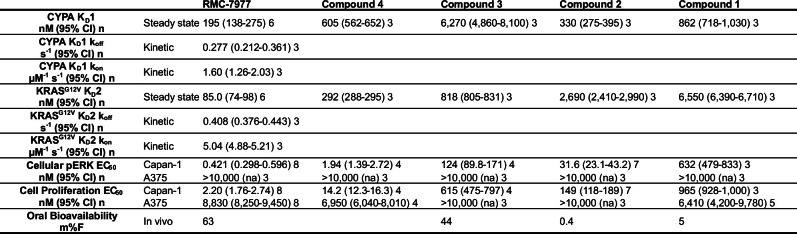
Compound potencies and properties

Potencies for compounds 1–4 and RMC-7977 in biophysical (K_D_1 and K_D_2 measured by SPR), biochemical (KRAS:BRAF RBD disruption measured by time-resolved fluorescence binding assay), cellular, and in vivo assays (RAS:CRAF RBD Disruption and RAS:CYPA Binding measured by nano-BRET, pERK measured by MSD, proliferation measured by CTG, Oral bioavailability measured by LC-MS/MS analysis, 0–24-hour AUC, of total blood exposure from a single oral dose compared to intravenous exposure). n=number of independent experiments performed.

**Extended Data Table 2:**
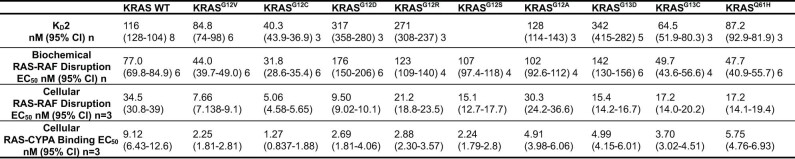
RMC-7977 KRAS potencies

Potencies for RMC-7977 in biophysical (steady state K_D_2 measured by SPR), biochemical (KRAS:BRAF RBD disruption measured by time-resolved fluorescence binding assay), and cellular assays (RAS:CRAF RBD disruption and RAS:CYPA Binding measured by nano-BRET). n=number of independent experiments performed.

**Extended Data Table 3:**
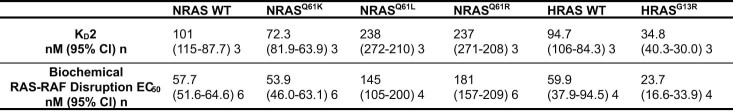
RMC-7977 NRAS and HRAS potencies

Potencies for RMC-7977 in biophysical (steady state K_D_2 measured by SPR), biochemical (KRAS:BRAF RBD disruption measured by time-resolved fluorescence binding assay). n=number of independent experiments performed.

## Data Availability

Source data have been provided for main and extended data figures. PDB files for all crystal structures are available through the PDB under accession numbers: 8TBF, 8TBG, 8TBH, 8TBI, 8TBJ, 8TBK, 8TBL, 8TBM and 8TBN. All other data and materials supporting the findings of this study are available in the main text or the supplementary materials. Source data are provided with this paper.
