# Supplementary material for: Concurrent inhibition of oncogenic and wild-type RAS-GTP for cancer therapy
Source: Nature. 2024 Apr 8;629(8013):919–26. doi: 10.1038/s41586-024-07205-6 (PMC11111408; doi:10.1038/s41586-024-07205-6)
Supplement: Supplementary file 1 — Supplementary Methods [file 41586_2024_7205_MOESM1_ESM.docx]

**Supplementary Methods**

**Tri-complex crystallization**

Purified human CYPA and RAS (untagged WT and mutants) bound to GMPPNP were combined in a 2:1 CYPA:RAS molar ratio in a buffer solution consisting of 12.5 mM HEPES-NaOH pH 7.3, 75 mM NaCl, 5 mM MgCl_2_. RMC-7977 was added from a 10 mM DMSO stock to give solutions of 100 µM RAS, 200 µM CYPA, and 300 µM RMC-7977 in 1 mL total volume. These mixtures were incubated for 5 minutes on ice and the tri-complexes were purified via gel filtration using a Superdex 75 10/300 GL column pre-equilibrated with a buffer consisting of 12.5 mM HEPES-NaOH pH 7.3, 75 mM NaCl, 5 mM MgCl_2_. Fractions containing the tri-complex were pooled and concentrated to 15 mg/mL using an Amicon Ultra-4 30K centrifugal filter (Millipore Sigma). 80 µL of a screen composed of 0.1 TRIS pH 8.0 and 20-30% PEG 4000 (increasing by 0.833% increments was dispensed into the wells of an MRC 2 crystallization plate. 0.3 µL of well solution was mixed with 0.3 µL of the concentrated tri-complex in a sitting drop and the plate was incubated at 18°C. Crystals grew overnight and were vitrified following cryoprotection via mother liquor supplemented with 12.5% glycerol.

**Crystallography data collection and refinement**

KRAS^G12A^, KRAS^G12C^, KRAS^G12R^, KRAS^WT^, HRAS^WT^, and NRAS^WT^ tri-complex X-ray diffraction datasets were collected at the Canadian Light Source (CLS CMCF-08ID, Saskatoon, Saskatchewan, Canada. Wavelength of 0.954 Å). The KRAS^G12S^ tri-complex X-ray diffraction dataset was collected at the National Synchrotron Light Source II (NSLS2 17-ID-2 (FMX), Upton, New York, USA. Wavelength of 0.979 Å). The KRAS^G12D^ tri-complex X-ray diffraction dataset was collected at the Advanced Light Source (ALS 5.0.1, Berkeley, California, USA. Wavelength of 0.977 Å). The KRAS^G12V^ tri-complex X-ray diffraction dataset was collected at the Stanford Synchrotron Radiation Lightsource (SSRL 12-2, Menlo Park, California, USA. Wavelength of 0.979 Å). Data collection and processing were performed as described previously.^1^ There are no Ramachandran outliers for any of the structures and 96.35% of residues or more fall in the favored region. Final processing and refinement statistics can be found in Supplementary Table 1.

**Cell culture and reagents**

The following cell lines were obtained from ATCC: AsPC-1, A375, CT26, Capan-1 HCT-116, Hs 766T, HuP-T3, KU1919, NCI-H1975, NCI-H358, NCI-H441, PSN1, SKMEL30, SW620, 293T, U2OS

**Effect of gene mutation status on *KRAS* dependence**

A Random Forest regression model was trained on the 22Q4 release of the DepMap somatic mutation and gene effect data^11^ to investigate the relationship between cell line gene mutation status and *KRAS, NRAS, or HRAS* dependence. The input features were an N x M matrix, where N is the number of cell lines and M is the number of genes that were mutated in at least 10% of the cell lines (N=1071, M=782). Each column is a binary representation of a gene’s mutation status for each cell line (1 if mutated, 0 if not). The target variable is a length-N 1-D vector of gene effect scores. The model consisted of 50 trees, and for each tree, 1/3 of the total number of features were considered at each split point.

The top 5 features with the largest feature importance values in the trained model were then tested for statistical significance. This was done by constructing 100 additional Random Forest regression models with randomly permuted effect scores and defining five null distributions consisting of the Nth largest feature importance value of each model across all 100 models, from N=1 to 5. A right-tailed hypothesis test was then performed for each feature with a Bonferroni-adjusted significance cutoff of p < 0.05 / 5 = 0.01. The top feature was compared to the N=1 distribution, the second feature was compared to the N=2 distribution, etc.

**PRISM assay**

*Associations between inhibitor sensitivity Area Under the Curve (AUC) and mutations*

Of the 931 cell lines assayed, AUC values were successfully derived for 869 cell lines by fitting a robust four-parameter logistic curve to the response of each cell line to the compound. For every gene with non-silent mutations in at least four cell-lines, we compared the AUC values between cells with and without those mutations using a t-test. This analysis was carried out for: (i) the full dataset; (ii) excluding cell lines with non-silent KRAS mutations; and (iii) excluding cell lines that have either KRAS or NRAS non-silent mutations.

*Bioinformatics analyses*

Gene mutation and gene expression data were downloaded from the 22Q4 release of the DepMap Data Portal^2^. All qc-filtered compound AUC values were cross-referenced with DepMap data using an exact matching of the cell line name. For tumor models with no publicly available data, we carried out whole exome sequencing to ascertain gene mutations and RNA sequencing to ascertain gene expression. DNA mutation calling was accomplished with TNSeq using the hg38 version of the human genome^3^. Functional annotation of the resulting mutation calls was accomplished with Variant Effect Predictor and further annotated with oncoKB^4^. Gene expression was quantified using salmon against the hg38 version of human transcriptome further processed using txImport and edgeR to generate normalized counts^5-7^.

**Western blot analysis**

Cells were lysed with MSD Tris Lysis Buffer (MSD, R60TX-2) or a buffer of 1% Triton X-100, 20 mM Tris-HCl, 150 mM NaCl, and 1 mM EDTA, both supplemented with protease and phosphatase inhibitors. Lysates were cleared by centrifugation and quantified by BCA assay (Pierce, 23225). Equal quantities of protein were resolved on 12% or 4-12% Bis-Tris polyacrylamide gels, then transferred to a nitrocellulose membrane using the iBlot 2.0 system or wet transfer, probed with primary and secondary antibodies, and detected by a LiCor Odyssey CLx imager using Image Studio (Version 5.2) or by chemiluminescence with Clarity or ClarityMax chemiluminescent substrates and a ChemiDoc XRS+ imager (Bio-Rad). Image Studio Lite (Version 5.2.5) and Image Lab (Version 6.1.0 build 7) software were used for data analysis.

Primary antibodies (all 1:1,000 dilution): anti-phospho-p44/42 MAPK (ERK1/2) T202/Y204 (cat # 9101; clone D13.14.4E, cat # 4370), anti-p44/42 (ERK1/2) (clone 3A7, cat # 9107; cat # 9102), anti-phospho-MEK1/2 S217/221 (cat # 9121), anti-MEK1/2 (clone L38C12, cat # 4694), anti-phospho-p90RSK S380 (clone D5D8, cat # 12032), anti-RSK1/RSK2/RSK3 (clone D7A2H, cat # 14813), anti-phospho-CRAF S338 (clone 56A6, cat # 9427), anti-CRAF (clone D5X6R, cat # 12552), anti-BIM (clone C34C5, cat # 2933), anti-PARP (clone 46D11, cat # 9532), anti-β-Actin (clone 8H10D10, cat # 3700), anti-vinculin (clone E1E9V, cat # 13901) from Cell Signaling Technology; anti-RAS (clone EPR3255, cat # ab108602) from Abcam; and anti-vinculin (clone hVIN-1, cat # V9131) from Millipore Sigma

LiCor secondary antibodies (1:20,000 dilution): Goat anti-rabbit IR800-conjugated (cat # 926-32211), goat anti-mouse IR800-conjugated (cat # 926-32210), goat anti-mouse IR680-conjugated (cat # 926-68070), and goat anti-rabbit IR680-conjugated (cat # 926-68071) from LiCor

HRP-linked secondary antibodies (1:2,000 dilution): anti-rabbit (cat # 7074) and anti-mouse (cat # 7076) from Cell Signaling Technology

**Xenograft studies**

Female BALB/c nude mice, NOD SCID and NSG mice 6-8 weeks old from Beijing Vital River/VR Laboratory Animal Co., LTD, Beijing AniKeeper Biotech Co., Ltd., Shanghai Sino-British SIPPR/BK Laboratory Animal Co., LTD and The Jackson Laboratory were used. Animals were housed in polycarbonate cages, in an environmentally monitored, well ventilated room maintained at a constant temperature of 20-26°C and relative humidity of 40-80%. Fluorescent lighting provided illumination approximately 12 hours per day. Each mouse was inoculated at the right flank with tumor cells (2x10^6^-1x10^7^) in 100-200 µL of media/PBS supplemented with Matrigel (1:1). Treatments were started when the average tumor size reached 100-250 mm^3^. Tumor size was measured at two dimensions using a digital caliper, and the tumor volume in mm^3^ was calculated using the formula: Volume = ((width)2 × length)/2. Mice on studies were weighed and tumor measurements collected twice weekly. Human primary cancer xenograft models were generated using fresh cancer tumor fragments obtained from hospitals with informed consent from the patients in accordance with protocols approved by the Hospital Institutional Ethical Committee (IEC). Tumor fragments were serial-passaged in BABL/c nude mice and then cryopreserved. Cryopreserved tumors were recovered by implantation in BALB/c nude mice and further serial passaged and frozen for study implantation use. Recovered tumor fragments of about 15-30 mm3 in size from each model were implanted into right flanks of Balb/c nude mice. Treatment started when average tumor volume reached 100-250mm^3^. **For efficacy study,** Animals were assigned to study groups using stratified randomization based upon their tumor volumes (n=3-10/group). RMC-7977 was administered via oral gavage at 10 mg/kg, using the formulation made of 10/20/10/60 (%v/v/v/v) DMSO/PEG 400/Solutol HS15/water; vehicle formulation was used for the control group. Treatments were administered daily for 7 days per week except for CRC G12V, LUAD G12D, CRC G12S, LUAD G12C, and PDAC G12D, which were all treated once daily for 5 days of treatment followed by 2 days of treatment cessation every week. Animals were treated for 28 days (or up to 110 for selected models) or an earlier time if tumor burden reached humane endpoint. Mean ± s.e.m was plotted in the waterfall plots. The dashed line on waterfall plot indicates a 10% reduction in tumor volume from baseline. Xenograft models with mean tumor volume reduction more than 10% will be considered models with regression upon RMC-7977 treatment. Body weights were collected twice a week during the study. **For single-dose pharmacokinetic/pharmacodynamics (PK/PD) study**, mice were randomized into treatment groups when mean tumor volume reached 400-600 mm^3^. At the indicated time points after a single dose of RMC-7977 at the indicated dose levels, blood and tumor samples were collected for PK and PD analysis, respectively.

**Sotorasib resistance xenograft study**

6-week-old female NSG mice (Charles River) were used. Tumor cells derived from a NSCLC patient who relapsed on treatment with sotorasib were injected subcutaneously in the flanks of the abdomen (2x10^6^ cells in 100µl of PBS) and formed palpable and measurable tumors after approximately 2 weeks. Tumor volume was monitored every other day using caliper, and the tumor volume in mm^3^ was calculated using the formula: Volume = ((width)^2^ × length)/2, where width and length are measured in mm. Compounds were administered by oral gavage and prepared as follows: sotorasib (50 mg/kg) was formulated in 2% (w/v) HPMC (Hydroxypropyl-methyl cellulose - Sigma H8384/ (v/v) 1% Tween 80; RMC-7977 (10 mg/kg) was formulated as described in Xenograft Studies.

**OVA peptide vaccination**

Bone marrow dendritic cell (BMDC) differentiation was previously described^8^. Briefly, bone marrow was isolated from the femurs and tibias of 6-8 week-old female C57BL/6J mice (Jackson Laboratory). Bone marrow cells were plated in complete RPMI containing 10% FBS, 1 mM Sodium Pyruvate, mM HEPES, 1x MEM Non-Essential Amino Acids, and supplemented with GMCSF (Peprotech) at 20 ng/ml. On day 10, 0.5 ng/ml IL-4 (Peptotech) was added to the media. 50% of the media was replaced with fresh GMCSF-containing media on culture days 6, 8, and 10. On day 11, 75% of media was replaced with fresh GMCSF- and IL-4-containing media. 100% of the media was replaced on culture days 12 and 13. To activate BMDCs prior to vaccination, 0.5 μg/ml CpG (Invivogen, tlrl-1668) and 1 μg/ml LPS (Sigma Aldrich, L5418) were added to the media on day 13. On culture day 14, activated BMDCs were pulsed with OVA peptide (SIINFEKL) (1 μg/ml, Anaspec, AS-60193-1) for 2 hours and washed 3 times in serum-free RPMI. 1x10^6^ peptide-pulsed, activated BMDCS were injected intravenously into each mouse on day 0 and day 8. Mice were treated with RMC-7977 (25 mg/kg) or vehicle PO daily starting one day before vaccination (day -1). After 15 days of treatment, mice were euthanized and spleens were harvested. Spleens were mechanically dissociated into single-cell suspension, incubated at 4°C for 20 minutes with Mouse Fc blocking agent (anti-CD16/32, Biolegend) and Live/Dead blue Viability Dye (ThermoFisher), and 20 minutes with cell staining antibody mix. Antibodies used were CD45 BUV395 1:800 (clone 30-F11, cat # 564279) and CD19 BUV661 1:400 (clone 1D3, cat # 612971) from BD Biosciences; CD8a FITC 1:400 (clone KT15, MA5-16759) from ThermoFisher; CD4 Spark NIR686 1:400 (clone GK1.5, cat # 100476) and CD3 ε APC/Fire750 1:400 (clone 145-2C11, cat # 100362) from BioLegend; and H-2K^b^ SIINFEKL tetramer PE 1:10 (cat # TB-5001-1) from MBL International Corporation. Samples were run on the Cytek Aurora flow cytometer and analyzed using the FlowJo software (Version 10.10) (BD Bioscience). Murine IFN𝛄 ELISpot (ImmunoSpot, CTL) was performed according to manufacturer’s protocol. Briefly, spleens were mechanically dissociated into single-cell suspension. 2x10^5^ splenocytes per well were left unstimulated, stimulated with 1 μg/ml SIINFEKL peptide (AnaSpec, cat # AS-60193-1), or 1 μg/ml each anti-CD3 (clone 145-2C11, BD Biosciences, cat # 553058) and anti-CD28 (clone 37.51, BD Biosciences, cat # 557393) antibodies. Plates were incubated at 37°C, 5% CO_2_ for 24 hours before staining and analysis on the CTL ImmunoSpot S6 Universal M2 Analyzer.

**Immune cell response *in vivo***

6–8-week-old female BALB/c immunocompetent mice (Jackson Laboratory) were inoculated subcutaneously at the right flank with 5x10^6^ eCT26 (KRAS^G12C/G12C^, ABCB1^-/-^) clone I20 cancer cells. Tumor width and length were measured with digital calipers, and tumor volume in mm^3^ was calculated using the formula: Volume = (width)^2^ × length)/2, where width and length are measured in mm. Body weight and tumor measurements were collected twice a week. After 4 days of dosing with vehicle or RMC-7977, tumor tissue was collected 24 hours post the last dose and processed for flow cytometric analysis. Tumor tissue was minced, processed with the Dri Tumor & Tissue Dissociation Reagent from BD Biosciences and homogenized with the gentleMACS^™^ Dissociator. Tumor cell suspensions were incubated at 4°C for 30 minutes with Mouse BD Fc Block (Clone 2.4G2 from BD Pharmingen), 10 minutes with Blue Dead Cell Stain Kit (Invitrogen) and 30 min in cell staining buffer. Antibodies used were CD45 BUV395 1:800 (Clone 30‑F11, cat # 565967), CD19 BUV661 1:400 (Clone 1D3, cat # 612971), and CD8b BV650 1:400 (Clone H35-17.2, cat # 740552) from BD Biosciences; and CD3ε APC/Fire750 1:400 (Clone [145-2C11](https://www.biolegend.com/en-us/search-results?Clone=145-2C11), cat # 100361), CD4 Spark NIR686 1:400 (Clone GK1.5, cat # 100475), and CD11b BV750 1:800 (Clone M1/70, cat # 101267) from Biolegend; MuLV gp70 (AH1) Tetramer reagent 1:20 from MBL International Corporation (cat # TB-M521-1) was used for tumor specific antigen staining. Cells were analyzed on a 4-laser Cytek Aurora (Cytek Biosciences), and data analysis was done using SpectroFlo (Cytek Biosciences) and FlowJo (FlowJo LLC.).

**References**

1 Schulze, C. J. *et al.* Chemical remodeling of a cellular chaperone to target the active state of mutant KRAS. *Science* **381**, 794-799 (2023). <https://doi.org:10.1126/science.adg9652>

2 Ghandi, M. *et al.* Next-generation characterization of the Cancer Cell Line Encyclopedia. *Nature* **569**, 503-508 (2019). <https://doi.org:10.1038/s41586-019-1186-3>

3 Freed, D. A., R.; Weber, J. A.; Edwards, J. S. The Sentieon Genomics Tools - A fast and accurate solution to variant calling from next-generation sequence data. *bioRxiv* (2017).

4 Chakravarty, D. *et al.* OncoKB: A Precision Oncology Knowledge Base. *JCO Precis Oncol* **2017** (2017). <https://doi.org:10.1200/PO.17.00011>

5 Patro, R., Duggal, G., Love, M. I., Irizarry, R. A. & Kingsford, C. Salmon provides fast and bias-aware quantification of transcript expression. *Nat Methods* **14**, 417-419 (2017). <https://doi.org:10.1038/nmeth.4197>

6 Robinson, M. D., McCarthy, D. J. & Smyth, G. K. edgeR: a Bioconductor package for differential expression analysis of digital gene expression data. *Bioinformatics* **26**, 139-140 (2010). <https://doi.org:10.1093/bioinformatics/btp616>

7 Soneson, C., Love, M. I. & Robinson, M. D. Differential analyses for RNA-seq: transcript-level estimates improve gene-level inferences. *F1000Res* **4**, 1521 (2015). <https://doi.org:10.12688/f1000research.7563.2>

8 Jin, D. & Sprent, J. GM-CSF Culture Revisited: Preparation of Bulk Populations of Highly Pure Dendritic Cells from Mouse Bone Marrow. *J Immunol* **201**, 3129-3139 (2018). <https://doi.org:10.4049/jimmunol.1800031>
