# Supplementary material for: Concurrent inhibition of oncogenic and wild-type RAS-GTP for cancer therapy
Source: Nature. 2024 Apr 8;629(8013):919–26. doi: 10.1038/s41586-024-07205-6 (PMC11111408; doi:10.1038/s41586-024-07205-6)
Supplement: Supplementary file 2 — Reporting Summary [file 41586_2024_7205_MOESM2_ESM.pdf]

nature portfolio

Corresponding author(s): Mallika Singh, David Wildes, Jacqueline A.M. Smith

Last updated by author(s): Jan 22, 2024

Reporting Summary

Nature Portfolio wishes to improve the reproducibility of the work that we publish. This form provides structure for consistency and transparency in reporting. For further information on Nature Portfolio policies, see our Editorial Policies and the Editorial Policy Checklist.

Statistics

For all statistical analyses, confirm that the following items are present in the figure legend, table legend, main text, or Methods section.

n/a	Confirmed	
☐	☒	The exact sample size (*n*) for each experimental group/condition, given as a discrete number and unit of measurement
☐	☒	A statement on whether measurements were taken from distinct samples or whether the same sample was measured repeatedly
☐	☒	The statistical test(s) used AND whether they are one- or two-sided<br>*Only common tests should be described solely by name; describe more complex techniques in the Methods section.*
☒	☐	A description of all covariates tested
☐	☒	A description of any assumptions or corrections, such as tests of normality and adjustment for multiple comparisons
☐	☒	A full description of the statistical parameters including central tendency (e.g. means) or other basic estimates (e.g. regression coefficient) AND variation (e.g. standard deviation) or associated estimates of uncertainty (e.g. confidence intervals)
☐	☒	For null hypothesis testing, the test statistic (e.g. *F*, *t*, *r*) with confidence intervals, effect sizes, degrees of freedom and *P* value noted<br>*Give P values as exact values whenever suitable.*
☒	☐	For Bayesian analysis, information on the choice of priors and Markov chain Monte Carlo settings
☒	☐	For hierarchical and complex designs, identification of the appropriate level for tests and full reporting of outcomes
☐	☒	Estimates of effect sizes (e.g. Cohen's *d*, Pearson's *r*), indicating how they were calculated

Our web collection on statistics for biologists contains articles on many of the points above.

Software and code

Policy information about availability of computer code

Data collection

Western blots: Image Studio (v.5.2) was used for collection

Data analysis

PRISM panel: Data manipulation and analysis were carried out using python (v.3.10.9), specifically pandas (v.1.5.3) for data import; sckit-learn (v. 1.2.1) for random forest building; matplotlib (v.3.7.1) for data visualization; numpy (v.1.23.5) for numerical calculations and; scipy (v.1.10.1) to carry out Wilcoxon rank sums tests. Additional analyses were carried out using R (v.4.1.2).

Crystallography: All data were processed with XDS, and initial structures were determined via Phaser using previously solved KRAS and CYPA as molecular replacement search models. Ligand restraints were generated using AceDRG5. The final structures were determined through iterative rounds of model building using Coot and refinement using REFMAC5 from the CCP4 suite and phenix.refine.

Western blots: Image Studio Lite v.5.2.5) and Image Lab (v.6.1.0 build 7) were used for analysis

Inhibitor response modeling, curve fitting, generation of graphs: Prism 9 (GraphPad) was used to plot data, estimate EC50 or IC50, and display data in graphical form.

Flow cytometry: Data was acquired using SpectroFlo (version 3.1.2) and analyzed with FlowJo (version 10.10)

For manuscripts utilizing custom algorithms or software that are central to the research but not yet described in published literature, software must be made available to editors and reviewers. We strongly encourage code deposition in a community repository (e.g. GitHub). See the Nature Portfolio guidelines for submitting code & software for further information.

Data

Policy information about availability of data

All manuscripts must include a data availability statement. This statement should provide the following information, where applicable:

- Accession codes, unique identifiers, or web links for publicly available datasets
- A description of any restrictions on data availability
- For clinical datasets or third party data, please ensure that the statement adheres to our policy

The data from this study are available from the corresponding authors upon reasonable request. NEED TO UPDATE

Research involving human participants, their data, or biological material

Policy information about studies with human participants or human data. See also policy information about sex, gender (identity/presentation), and sexual orientation and race, ethnicity and racism.

Reporting on sex and gender	N/A
Reporting on race, ethnicity, or other socially relevant groupings	N/A
Population characteristics	N/A
Recruitment	N/A
Ethics oversight	N/A

Note that full information on the approval of the study protocol must also be provided in the manuscript.

Field-specific reporting

Please select the one below that is the best fit for your research. If you are not sure, read the appropriate sections before making your selection.

☒ Life sciences ☐ Behavioural & social sciences ☐ Ecological, evolutionary & environmental sciences

For a reference copy of the document with all sections, see nature.com/documents/nr-reporting-summary-flat.pdf

Life sciences study design

All studies must disclose on these points even when the disclosure is negative.

Sample size	Sample sizes for animal studies were determined based on statistical power analysis devised to detect significant differences of 30% tumor growth inhibition when possible. For some studies, n=3 mice were used per group. For in vitro studies, three or more identical wells of each treatment condition (biological replicates) were included in a single experiment when possible. In the event of limiting reagents, a minimum of two biological replicates were included within an experiment. Many experiments with only two biological replicates involved concentration response curves, and curve fit also increases confidence in individual data points.
Data exclusions	Aside from obvious experimental errors, no data were excluded from the analyses.
Replication	Most in vitro studies were repeated in three or more independent experiments or as noted in figure legends. Independent experiments were repeated over the course of several weeks or months. In vivo replicates were included in the data shown. Figures show representative data, and source data for all replicates have been provided.
Randomization	Tumor-bearing animals in efficacy studies were subject to block randomization resulting in equally sized groups with the same mean tumor volumes.
Blinding	Data acquisition in animal studies was carried out by different researchers than those that carried out data analysis. Investigators performing animal dosing and handling were only aware that the compounds were for the RAS MULTI program at Revolution Medicines, whereas the investigators writing the reports were not necessarily blinded to the identity of the compounds.

Reporting for specific materials, systems and methods

We require information from authors about some types of materials, experimental systems and methods used in many studies. Here, indicate whether each material, system or method listed is relevant to your study. If you are not sure if a list item applies to your research, read the appropriate section before selecting a response.

Materials & experimental systems

n/a	Involved in the study
☐	☒ Antibodies
☐	☒ Eukaryotic cell lines
☒	☐ Palaeontology and archaeology
☐	☒ Animals and other organisms
☒	☐ Clinical data
☒	☐ Dual use research of concern
☒	☐ Plants

Methods

n/a	Involved in the study
☒	☐ ChIP-seq
☐	☒ Flow cytometry
☒	☐ MRI-based neuroimaging

Antibodies

Antibodies used

The following primary antibodies were used at 1:1,000 dilution: anti-phospho-p44/42 MAPK (ERK1/2) T202/Y204 (no. 9101; clone D13.14.4E, no. 4370), anti-p44/42 (ERK1/2) (clone 3A7, no. 9107; no. 9102), anti-phospho-MEK1/2 S217/221 (no. 9121), anti-MEK1/2 (clone L38C12, no. 4694), anti-phospho-p90RSK S380 (clone D5D8, no. 12032), anti-RSK1/RSK2/RSK3 (clone D7A2H, no. 14813), anti-phospho-CRAF S338 (clone 56A6, no. 9427), anti-CRAF (clone D5X6R, no. 12552), anti-BIM (clone C34C5, no. 2933), anti-PARP (clone 46D11, no. 9532), anti-β-Actin (clone 8H10D10, no. 3700), anti-vinculin (clone E1E9V, no. 13901) all from Cell Signaling Technology; anti-RAS (clone EPR3255, no. ab108602) from Abcam; and anti-vinculin (clone hVIN-1, no. V9131) from Millipore Sigma.

The following secondary antibodies were used: goat anti-rabbit IR800-conjugated secondary (LiCor, no. 926-32211), goat anti-mouse IR800-conjugated secondary (LiCor, no. 926-32210), goat anti-mouse IR680-conjugated (LiCor, no. 926-68070), and goat anti-rabbit IR680-conjugated (LiCor, no. 926-68071) secondary antibodies at 1:20,000 dilution; and HRP-linked anti-rabbit (Cell Signaling Technology, no. 7074) and HRP-linked anti-mouse (Cell Signaling Technology, no. 7076) secondary antibodies at 1:2,000.

Validation

All antibodies used were validated by the respective commercial source for the application used in this manuscript.

Cell Signaling Technologies: "Validation steps include: 1) Examination of several cell lines and/or tissues of known expression levels allows accurate determination of species cross-reactivity and verifies specificity. 2) Treatment of cell lines with growth factors, chemical activators or inhibitors, which induce or inhibit target expression, verifies specificity. Phosphatase treatment confirms phospho-specificity. 3) The use of siRNA transfection or knockout cell lines verifies target specificity. 4) Side-by-side comparison of lots to ensures lot-to-lot consistency. 5) Optimal dilutions and buffers are predetermined, positive and negative cell extracts are specified, and detailed protocols are already optimized, saving valuable time and reagents."

Abcam: "Antibodies are validated in western blot using lysates from cells or tissues that we have identified to express the protein of interest. Once we have determined the right lysates to use, western blots are run and the band size is checked for the expected molecular weight. We will always run several controls in the same western blot experiment, including positive lysate and negative lysate (if possible). When possible, we also include knock-out (KO) cell lines as a true negative control for our western blots. We are always increasing the number of KO-validated antibodies we provide. In addition, we run old stock alongside our new stock. If we know the old stock works well, this also acts as a suitable positive control. If the western blot result gives a clear clean band and we are happy with the result from the control lanes, these antibodies will be passed and added to the catalog."

Millipore-Sigma: "WB validation is performed using multiple cell lysates or tissue lysates to explore the range of detectable protein expression in various tissues and species. At EMD Millipore, each antibody we develop is tested using an extensive internal cell bank and lysate library representing diverse growth conditions and treatments. The library of available test samples houses thousands of different cells, tissue lysates and blots, all of which have been tested and QC-controlled. This collection of testing materials provides us with a consistent and reliable source of high-quality testing material for our stringent WB validation studies."

Eukaryotic cell lines

Policy information about cell lines and Sex and Gender in Research

Cell line source(s)

The following cell lines were used in the study: AsPC-1, A375, CT26, Capan-1 HCT-116, Hs 766T, HuP-T3, KU1919, NCI-H1975, NCI-H358, NCI-H441, PSN1, SKMEL30, SW620, 293T, and U2OS obtained from ATCC; Pa14C and Pa16C cells were provided as a gift by Anirban Maitra; "RAS-less" mouse embryonic fibroblast (MEF) cell lines were obtained from the NIH (NCI RAS initiative at the FNLCR); AsPC-1 and NCI-H441 CYPA KO cells were generated by Synthego; NCI-H358 cells expressing low and high levels of doxycycline-inducible CYPA were engineered by WarpDrive Bio; NCI-H358 cells overexpressing doxycycline-inducible full-length or fusion RTKs were engineered by Revolution Medicines. eCT26 KRAS G12C/G12C ABCB1-/- cells were engineered at Synthego. Cell lines included in cell panels were acquired by the Broad Institute (PRISM) and Crown Bioscience.

Authentication

All cell lines tested were authenticated by STR analysis.

Mycoplasma contamination

All cell lines tested were negative for mycoplasma contamination.

Commonly misidentified lines
(See ICLAC register)

No commonly misidentified lines were used.

Animals and other research organisms

Policy information about studies involving animals; ARRIVE guidelines recommended for reporting animal research, and Sex and Gender in Research

Laboratory animals	Female Balb/c nude mice at 6-8 weeks of age were implanted with tumor cells for xenograft studies. Patient xenografts with resistance to sotorasib were implanted into female NOD scid gamma (NSG) mice at 6 weeks of age. 6-8 week-old female C57BL/6J mice were used for OVA peptide vaccination studies. 6–8-week-old female BALB/c immunocompetent mice were implanted with eCT26 KRAS G12C/G12C ABCB1-/- cells to assess immune cell response in vivo.
Wild animals	N/A
Reporting on sex	Studies utilized female mice.
Field-collected samples	N/A
Ethics oversight	All CDX/PDX mouse efficacy and PK/PD studies and procedures related to animal handling, care and treatment were conducted in compliance with all applicable regulations and guidelines of the relevant Institutional Animal Care and Use Committee (IACUC). For the sotorasib resistance PDX studies, all experiments were performed in accordance with the guideline for Ethical Conduct in the Care and Use of Animals as stated in The International Guiding Principles for Biomedical Research Involving Animals, developed by the Council for International Organizations of Medical Sciences.

Note that full information on the approval of the study protocol must also be provided in the manuscript.

Flow Cytometry

Plots

Confirm that:

- [ ] The axis labels state the marker and fluorochrome used (e.g. CD4-FITC).
- [ ] The axis scales are clearly visible. Include numbers along axes only for bottom left plot of group (a 'group' is an analysis of identical markers).
- [ ] All plots are contour plots with outliers or pseudocolor plots.
- [ ] A numerical value for number of cells or percentage (with statistics) is provided.

Methodology

Sample preparation	For the OVA vaccination experiment single cells suspension of splenocytes was prepared by smashing the spleen with a syringe plunger and passing over a 40uM cell strainer. Red blood cells were lysed in ACK buffer. Tumor samples were prepared by mechanical tumor digestion using the GentleMACS (Myltenyi) following by enzymatic digestion with the Dri Tumor & Tissue Dissociation Reagent (BD Biosciences).
Instrument	Cytek Aurora Flow Cytometer
Software	Data was acquired using SpectroFlo software (version 3.1.2) and analyzed using FlowJo (version 10.10)
Cell population abundance	*Describe the abundance of the relevant cell populations within post-sort fractions, providing details on the purity of the samples and how it was determined.*
Gating strategy	*Describe the gating strategy used for all relevant experiments, specifying the preliminary FSC/SSC gates of the starting cell population, indicating where boundaries between "positive" and "negative" staining cell populations are defined.*

- [ ] Tick this box to confirm that a figure exemplifying the gating strategy is provided in the Supplementary Information.
