# Supplementary material for: Concurrent inhibition of oncogenic and wild-type RAS-GTP for cancer therapy
Source: Nature. 2024 Apr 8;629(8013):919–26. doi: 10.1038/s41586-024-07205-6 (PMC11111408; doi:10.1038/s41586-024-07205-6)
Supplement: Supplementary file 3 — Table of data collection and refinement statistics (molecular replacement) for crystal structures. [file 41586_2024_7205_MOESM3_ESM.docx]

**Supplementary Table 1** **Data collection and refinement statistics (molecular replacement)**

|  | KRAS^G12A^ | KRAS^G12C^ | KRAS^G12D^ | KRAS^G12R^ | KRAS^G12S^ |
| --- | --- | --- | --- | --- | --- |
| **Data collection*** |  |  |  |  |  |
| Space group | P 2_1_ 2_1_ 2_1_ | P 2_1_ 2_1_ 2_1_ | P 2_1_ 2_1_ 2_1_ | P 2_1_ 2_1_ 2_1_ | P 2_1_ 2_1_ 2_1_ |
| Cell dimensions |  |  |  |  |  |
| *a*, *b*, *c* (Å) | 65.45, 81.46, 127.23 | 65.47, 83.57, 126.21 | 65.95, 83.79, 127.06 | 66.42, 85.67, 127.53 | 65.78, 84.34, 126.63 |
| α, β, γ (°) | 90, 90, 90 | 90, 90, 90 | 90, 90, 90 | 90, 90, 90 | 90, 90, 90 |
| Resolution (Å)** | 1.45 (1.50-1.45) | 1.26 (1.31-1.26) | 1.88 (1.95-1.88) | 1.50 (1.55-1.50) | 1.46 (1.51-1.46) |
| *R*_merge_ | 0.104 (1.36) | 0.075 (2.09) | 0.174 (1.87) | 0.105 (2.69) | 0.086 (2.05) |
| *I* / σ*I* | 8.48 (1.18) | 11.03 (0.93) | 8.95 (0.98) | 7.51 (0.55) | 9.99 (0.99) |
| Completeness (%) | 99.94 (99.85) | 98.27 (95.69) | 98.61 (97.51) | 99.75 (99.25) | 98.38 (96.14) |
| Redundancy | 7.3 (7.6) | 7.6 (7.5) | 6.7 (6.4) | 7.3 (7.5) | 7.3 (7.3) |
|  |  |  |  |  |  |
| **Refinement** |  |  |  |  |  |
| Resolution (Å) | 1.45 | 1.26 | 1.88 | 1.50 | 1.46 |
| No. reflections | 120928 (11961) | 183467 (17699) | 57210 (5573) | 116906 (11564) | 120857 (11653) |
| *R*_work_ / *R*_free_ | 18.5/21.5 | 14.6/18.4 | 17.1/22.7 | 19.9/22.7 | 15.4/20.0 |
| No. atoms |  |  |  |  |  |
| Protein | 5312 | 5339 | 5272 | 5250 | 5282 |
| Ligand/ion | 200 | 190 | 190 | 190 | 190 |
| Water | 752 | 984 | 717 | 588 | 746 |
| *B*-factors |  |  |  |  |  |
| Protein | 23.98 | 23.43 | 29.92 | 34.99 | 30.09 |
| Ligand/ion | 17.56 | 16.50 | 21.77 | 26.50 | 19.68 |
| Water | 33.8 | 40.87 | 36.8 | 39.5 | 42.83 |
| R.m.s. deviations |  |  |  |  |  |
| Bond lengths (Å) | 0.016 | 0.005 | 0.007 | 0.006 | 0.016 |
| Bond angles (°) | 1.5 | 0.84 | 0.81 | 0.78 | 1.42 |

*Each dataset was obtained from a single crystal.

**Values in parentheses are for highest-resolution shell.

|  | KRAS^G12V^ | KRAS^WT^ | HRAS^WT^ | NRAS^WT^ |
| --- | --- | --- | --- | --- |
| **Data collection*** |  |  |  |  |
| Space group | P 2_1_ 2_1_ 2_1_ | P 2_1_ 2_1_ 2_1_ | P 2_1_ 2_1_ 2_1_ | P 2_1_ 2_1_ 2_1_ |
| Cell dimensions |  |  |  |  |
| *a*, *b*, *c* (Å) | 65.56, 84.09, 127.10 | 65.76, 84.24, 125.98 | 65.45, 80.86, 128.21 | 65.49 84.27 126.04 |
| α, β, γ (°) | 90, 90, 90 | 90, 90, 90 | 90, 90, 90 | 90, 90, 90 |
| Resolution (Å)** | 1.57 | 1.50 | 1.20 | 1.59 |
| *R*_merge_ | 0.106 (2.78) | 0.059 (0.389) | 0.0972 (1.63) | 0.119 (2.36) |
| *I* / σ*I* | 10.67 (0.93) | 17.10 (4.54) | 8.22 (0.82) | 8.84 (0.77) |
| Completeness (%) | 98.21 (97.67) | 99.81 (99.57) | 99.54 (97.18) | 99.90 (99.91) |
| Redundancy | 6.6 (6.9) | 7.3 (7.4) | 7.1 (4.8) | 7.4 (7.7) |
|  |  |  |  |  |
| **Refinement** |  |  |  |  |
| Resolution (Å) | 1.57 | 1.50 | 1.20 | 1.59 |
| No. reflections | 96907 (9506) | 112271 (11048) | 211361 (20454) | 94249 (9290) |
| *R*_work_ / *R*_free_ | 17.8/20.9 | 17.8/21.0 | 16.1/19.2 | 18.3/21.8 |
| No. atoms |  |  |  |  |
| Protein | 5244 | 5325 | 5351 | 5273 |
| Ligand/ion | 196 | 194 | 196 | 190 |
| Water | 630 | 939 | 851 | 738 |
| *B*-factors |  |  |  |  |
| Protein | 29.69 | 24.48 | 19.20 | 31.69 |
| Ligand/ion | 20.81 | 15.16 | 13.58 | 27.08 |
| Water | 37.70 | 33.87 | 31.92 | 40.74 |
| R.m.s. deviations |  |  |  |  |
| Bond lengths (Å) | 0.006 | 0.006 | 0.006 | 0.011 |
| Bond angles (°) | 0.84 | 0.91 | 0.87 | 1.14 |
