# Supplementary material for: Concurrent inhibition of oncogenic and wild-type RAS-GTP for cancer therapy
Source: Nature. 2024 Apr 8;629(8013):919–26. doi: 10.1038/s41586-024-07205-6 (PMC11111408; doi:10.1038/s41586-024-07205-6)
Supplement: Supplementary file 4 — Unprocessed western blots [file 41586_2024_7205_MOESM4_ESM.pptx]

## Slide 1
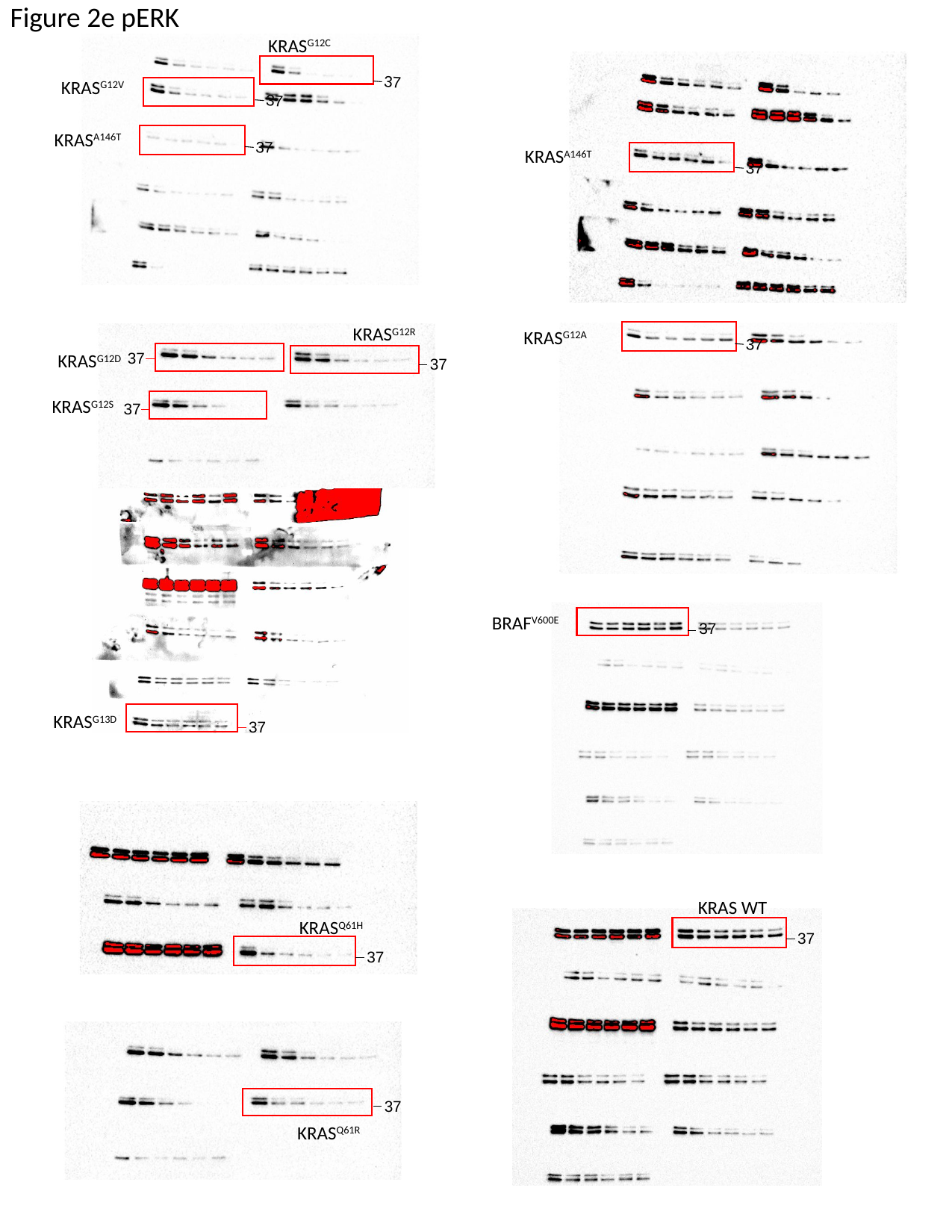

Figure 2e pERK
KRASG12C
KRASG12V
KRASA146T
KRASA146T
37
37
37
37
KRASG12R
KRASG12D
KRASG12S
KRASG12A
37
37
37
37
KRASG13D
BRAFV600E
37
37
KRASQ61H
KRAS WT
37
37
KRASQ61R
37

## Slide 2
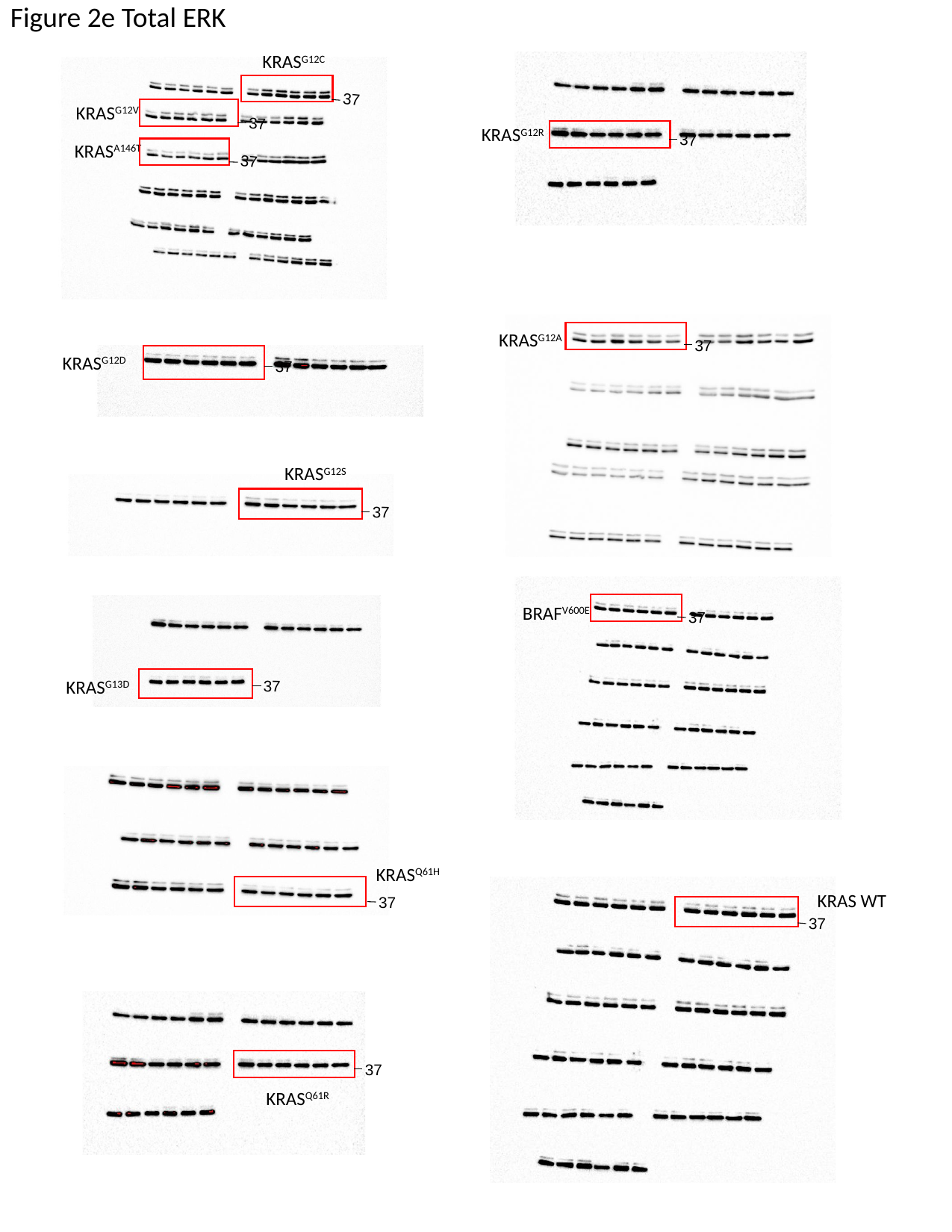

Figure 2e Total ERK
KRASG12C
KRASG12V
KRASA146T
KRASG12R
37
37
37
37
KRASG12A
37
KRASG12D
37
KRASG12S
37
BRAFV600E
KRASG13D
37
37
KRASQ61H
KRAS WT
37
37
KRASQ61R
37

## Slide 3
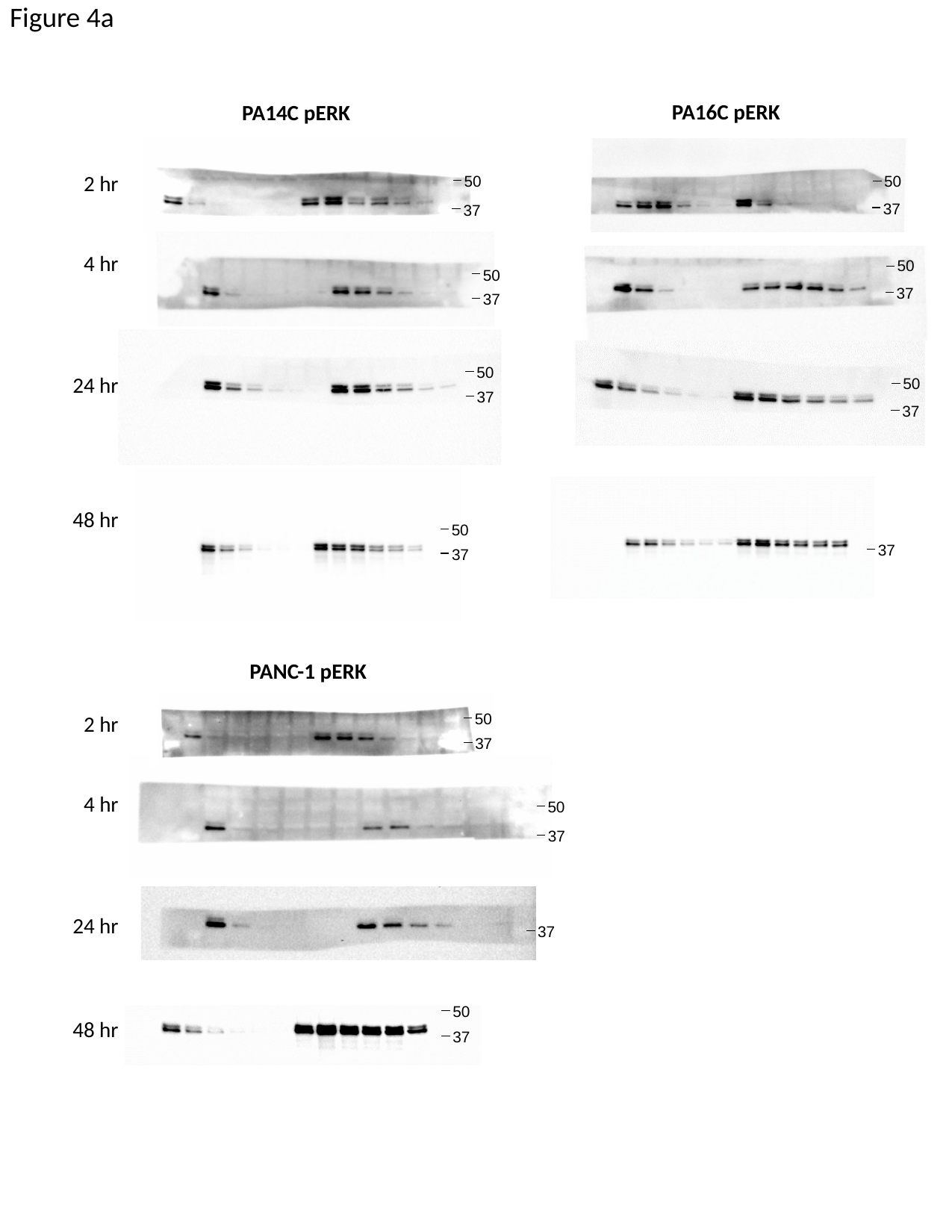

Figure 4a
PA16C pERK
PA14C pERK
2 hr
50
50
37
37
4 hr
50
50
37
37
50
24 hr
50
37
37
48 hr
50
37
37
PANC-1 pERK
50
2 hr
37
4 hr
50
37
24 hr
37
50
48 hr
37

## Slide 4
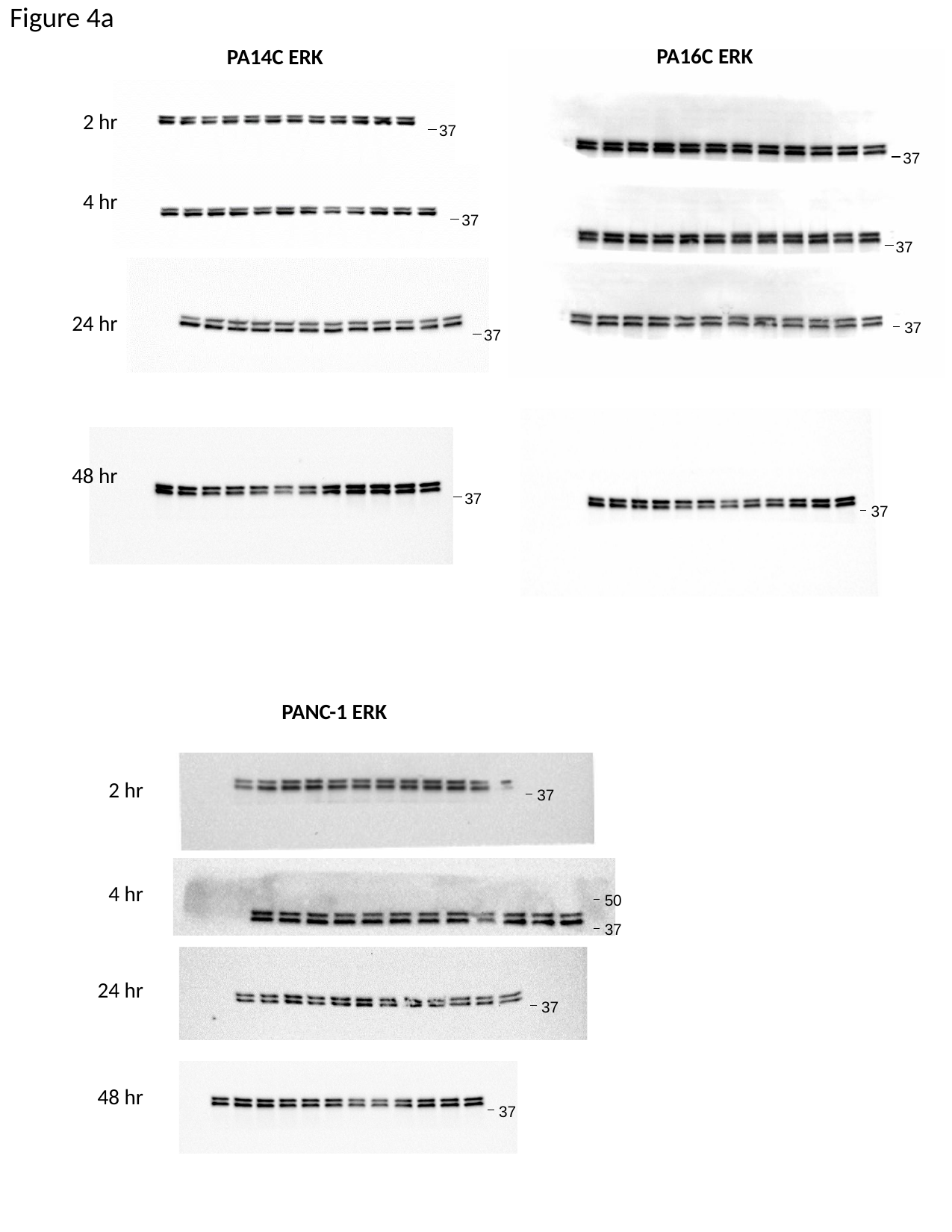

Figure 4a
PA16C ERK
PA14C ERK
2 hr
37
37
4 hr
37
37
24 hr
37
37
48 hr
37
37
PANC-1 ERK
2 hr
37
4 hr
50
37
24 hr
37
48 hr
37

## Slide 5
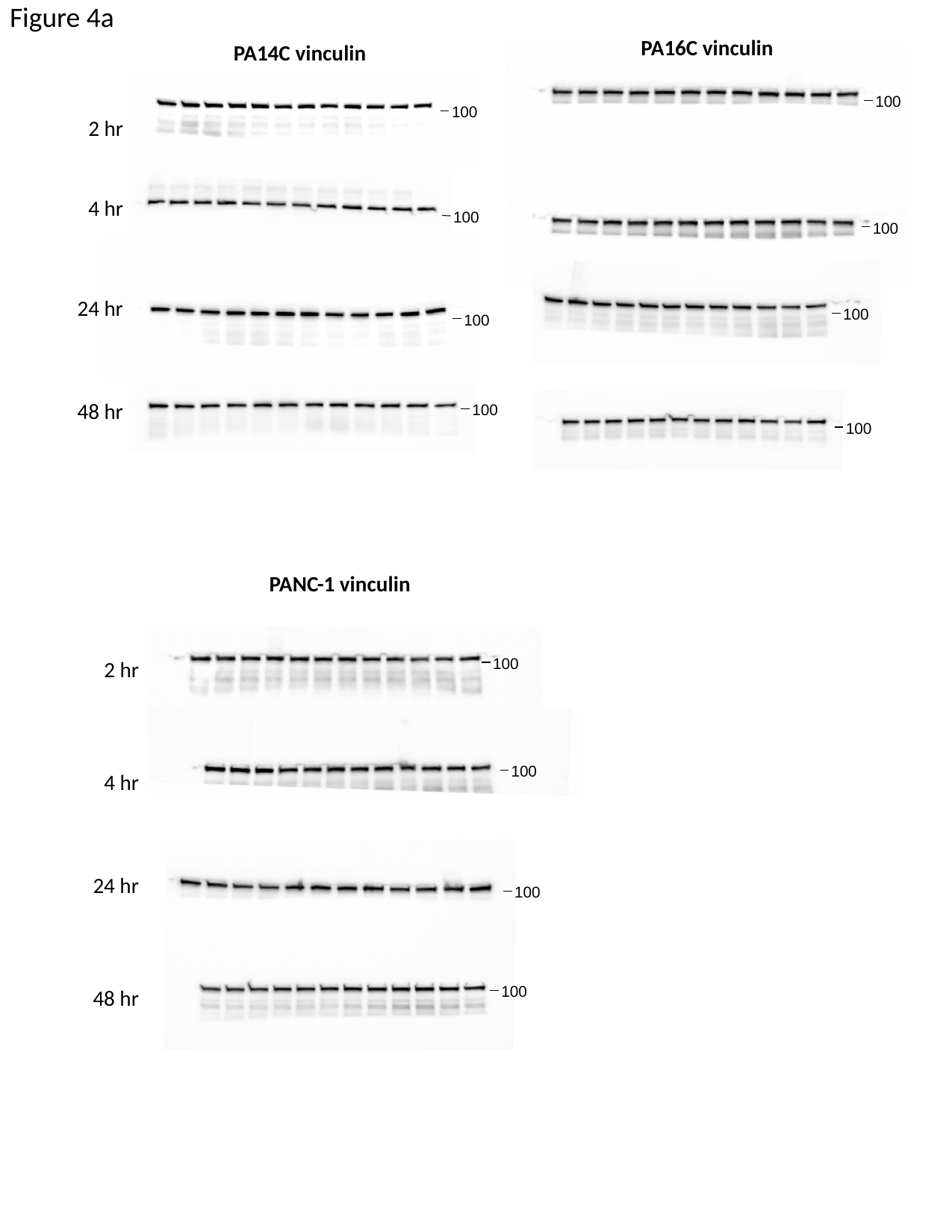

Figure 4a
PA16C vinculin
PA14C vinculin
100
100
2 hr
4 hr
100
100
24 hr
100
100
48 hr
100
100
PANC-1 vinculin
100
2 hr
100
4 hr
24 hr
100
100
48 hr

## Slide 6
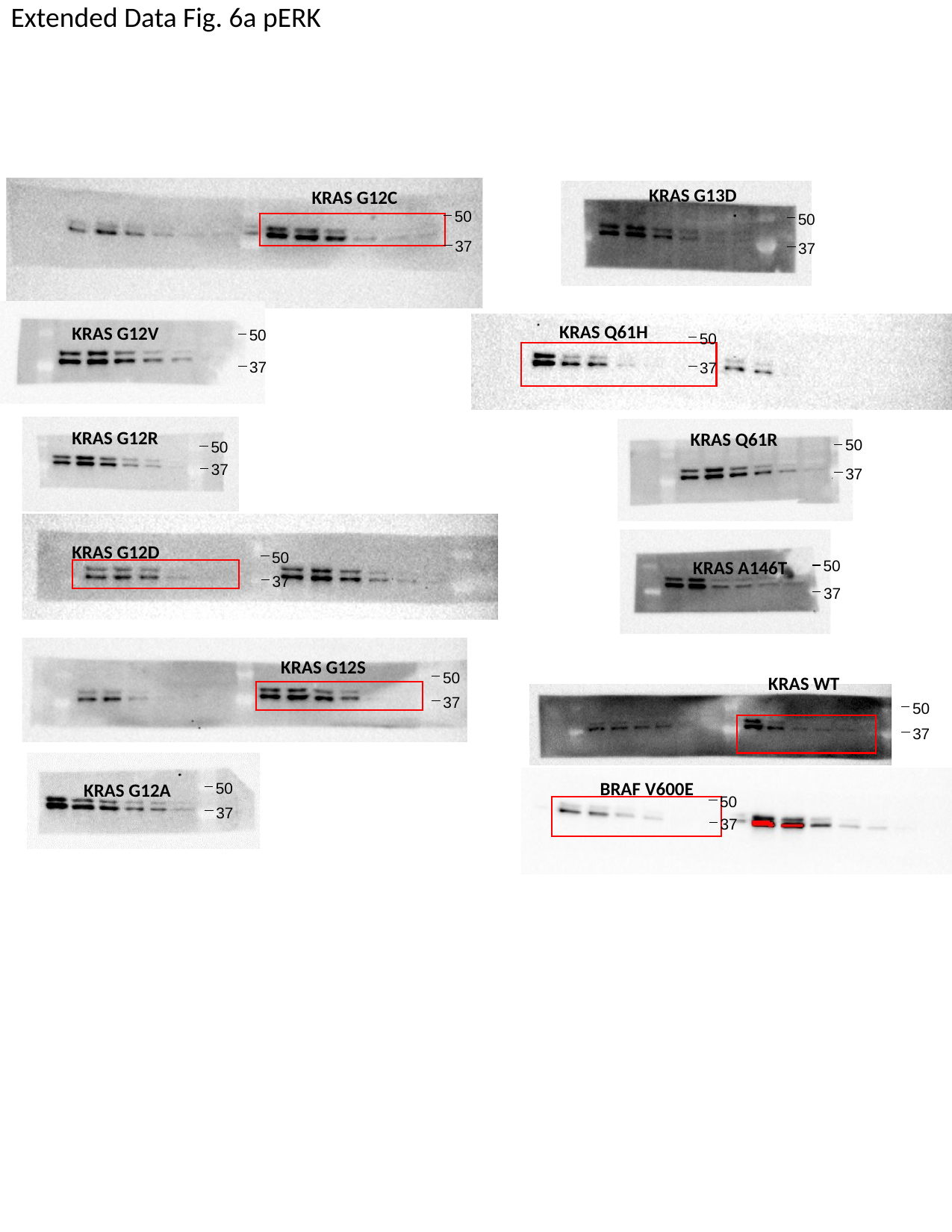

Extended Data Fig. 6a pERK
KRAS G13D
KRAS G12C
KRAS Q61H
KRAS G12V
KRAS G12R
KRAS Q61R
KRAS G12D
KRAS A146T
KRAS G12S
KRAS WT
BRAF V600E
KRAS G12A
50
50
37
37
50
50
37
37
50
50
37
37
50
50
37
37
50
37
50
37
50
50
37
37

## Slide 7
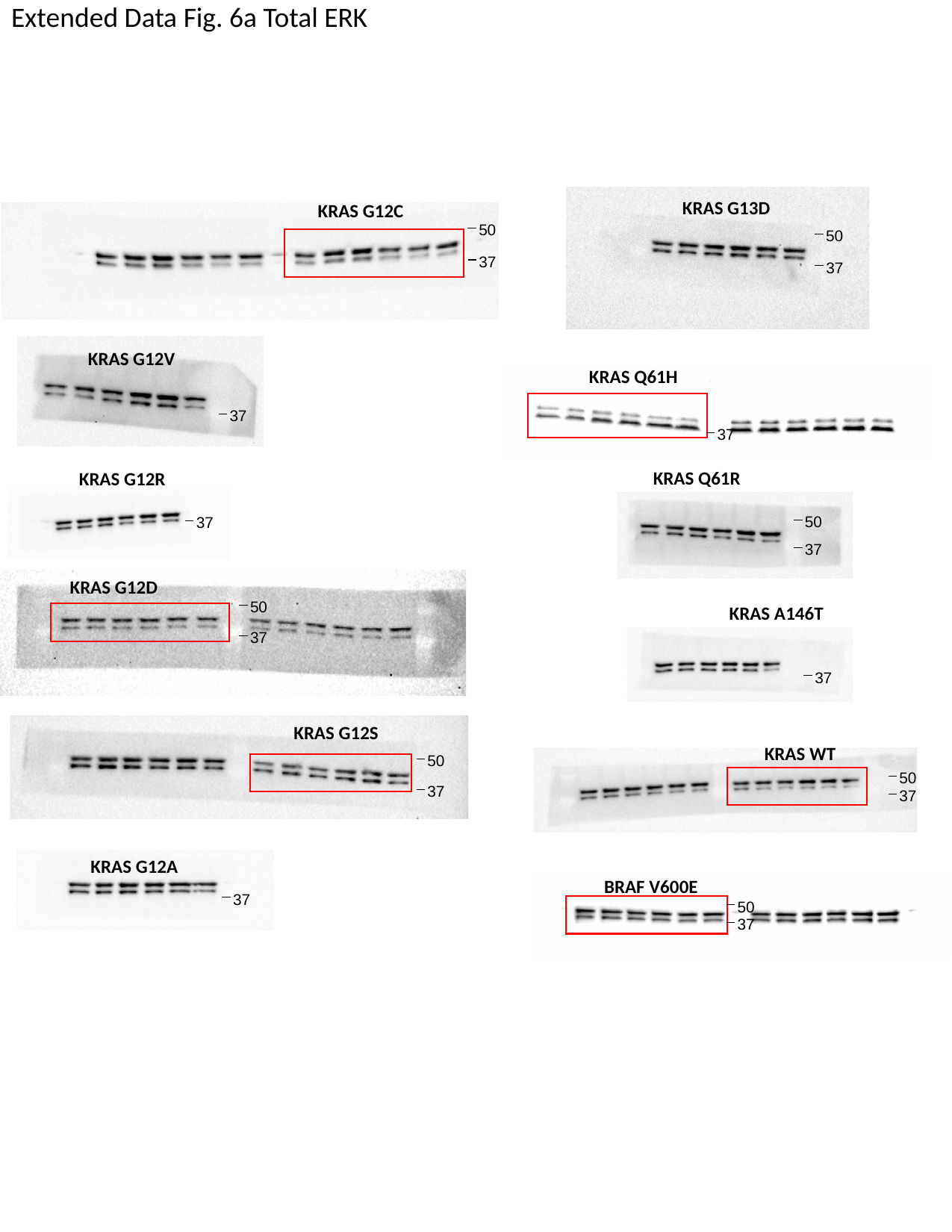

Extended Data Fig. 6a Total ERK
KRAS G13D
KRAS G12C
KRAS G12V
KRAS Q61H
KRAS Q61R
KRAS G12R
KRAS G12D
KRAS A146T
KRAS G12S
KRAS WT
KRAS G12A
BRAF V600E
50
50
37
37
37
37
50
37
37
50
37
37
50
50
37
37
37
50
37

## Slide 8
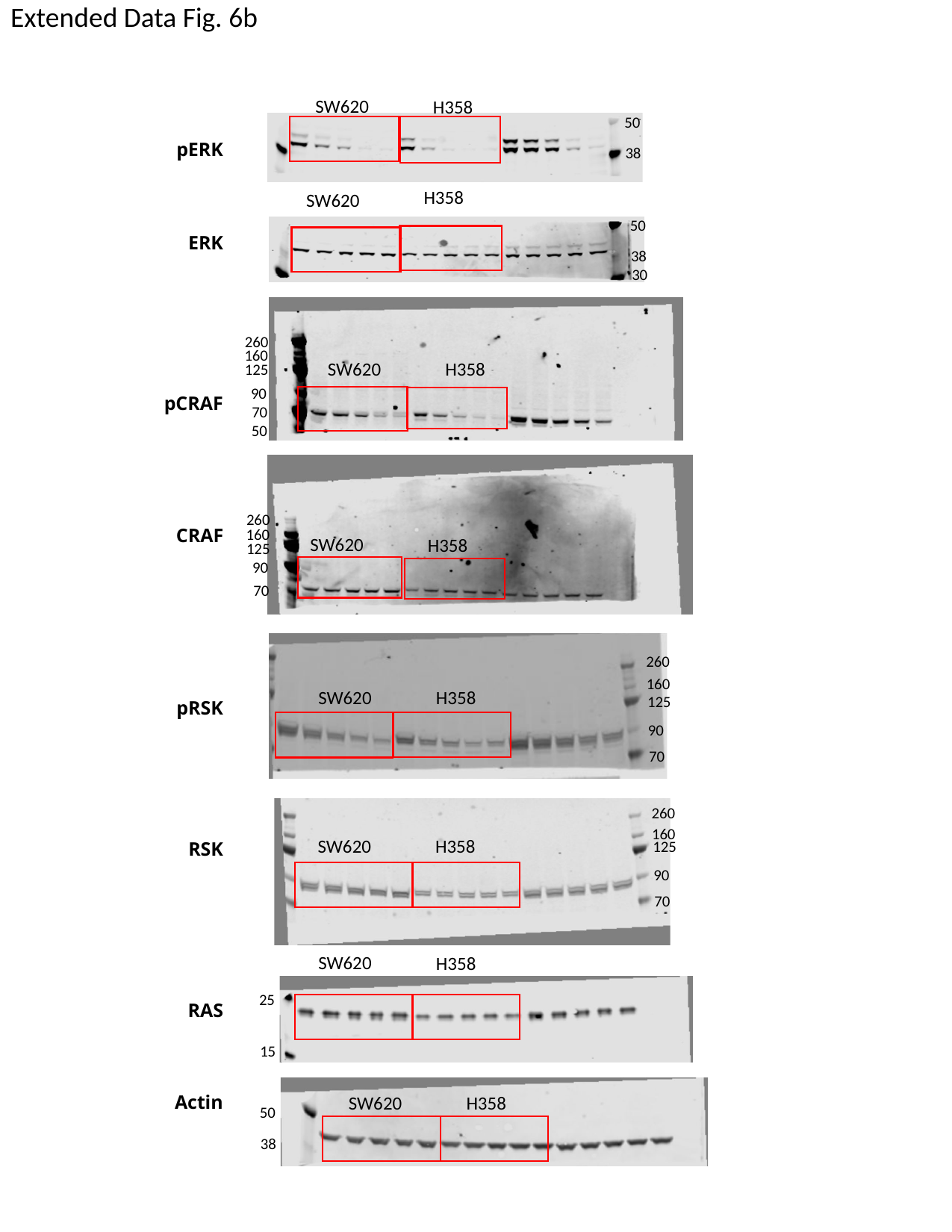

Extended Data Fig. 6b
SW620
H358
50
pERK
38
H358
SW620
50
ERK
38
30
260
160
SW620
H358
125
90
pCRAF
70
50
260
160
CRAF
SW620
H358
125
90
70
260
160
SW620
H358
125
pRSK
90
70
260
160
SW620
H358
125
RSK
90
70
SW620
H358
25
RAS
15
SW620
H358
Actin
50
38

## Slide 9
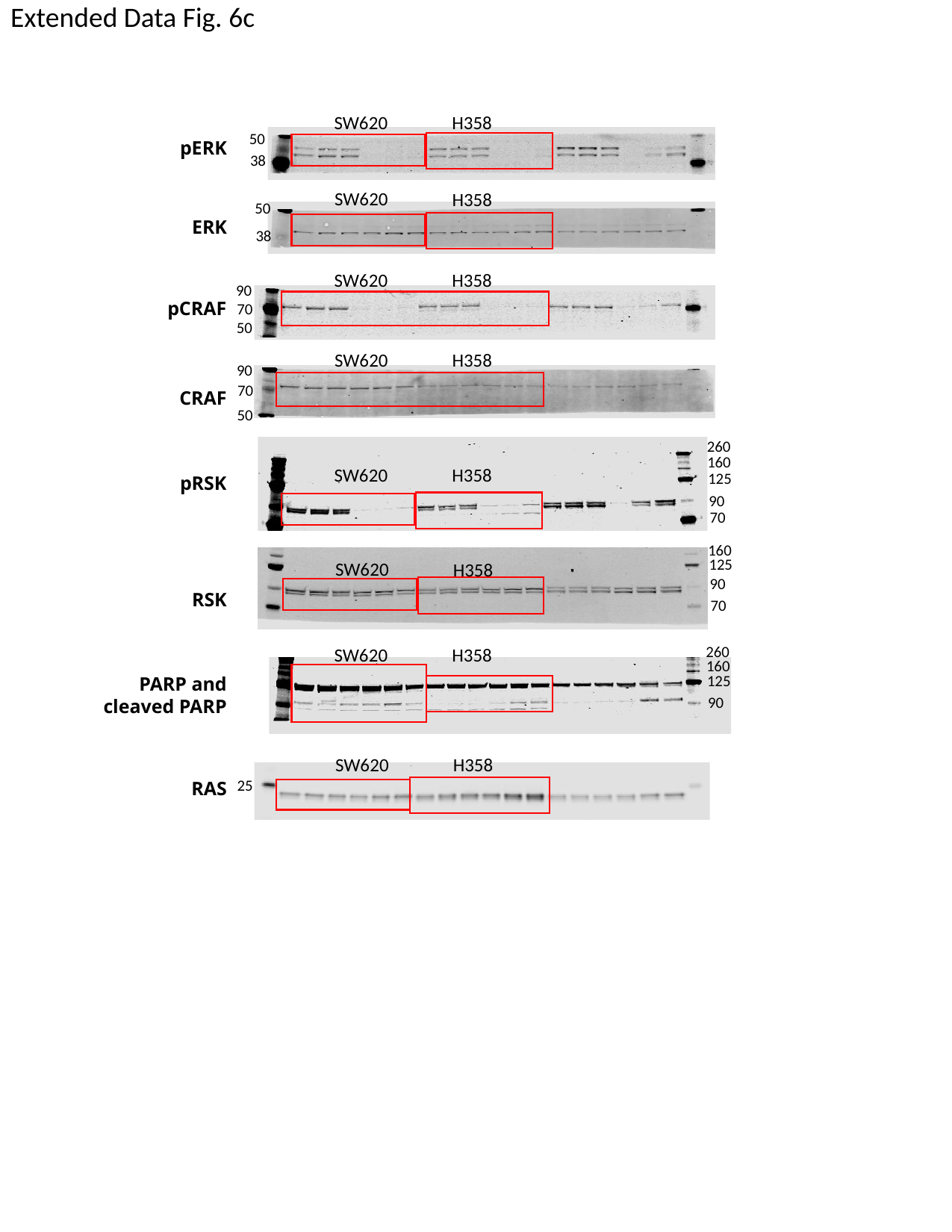

Extended Data Fig. 6c
SW620
H358
50
pERK
38
SW620
H358
50
ERK
38
SW620
H358
90
pCRAF
70
50
SW620
H358
90
70
CRAF
50
260
160
SW620
H358
125
pRSK
90
70
160
125
SW620
H358
90
RSK
70
260
SW620
H358
160
125
PARP and cleaved PARP
90
SW620
H358
25
RAS

## Slide 10
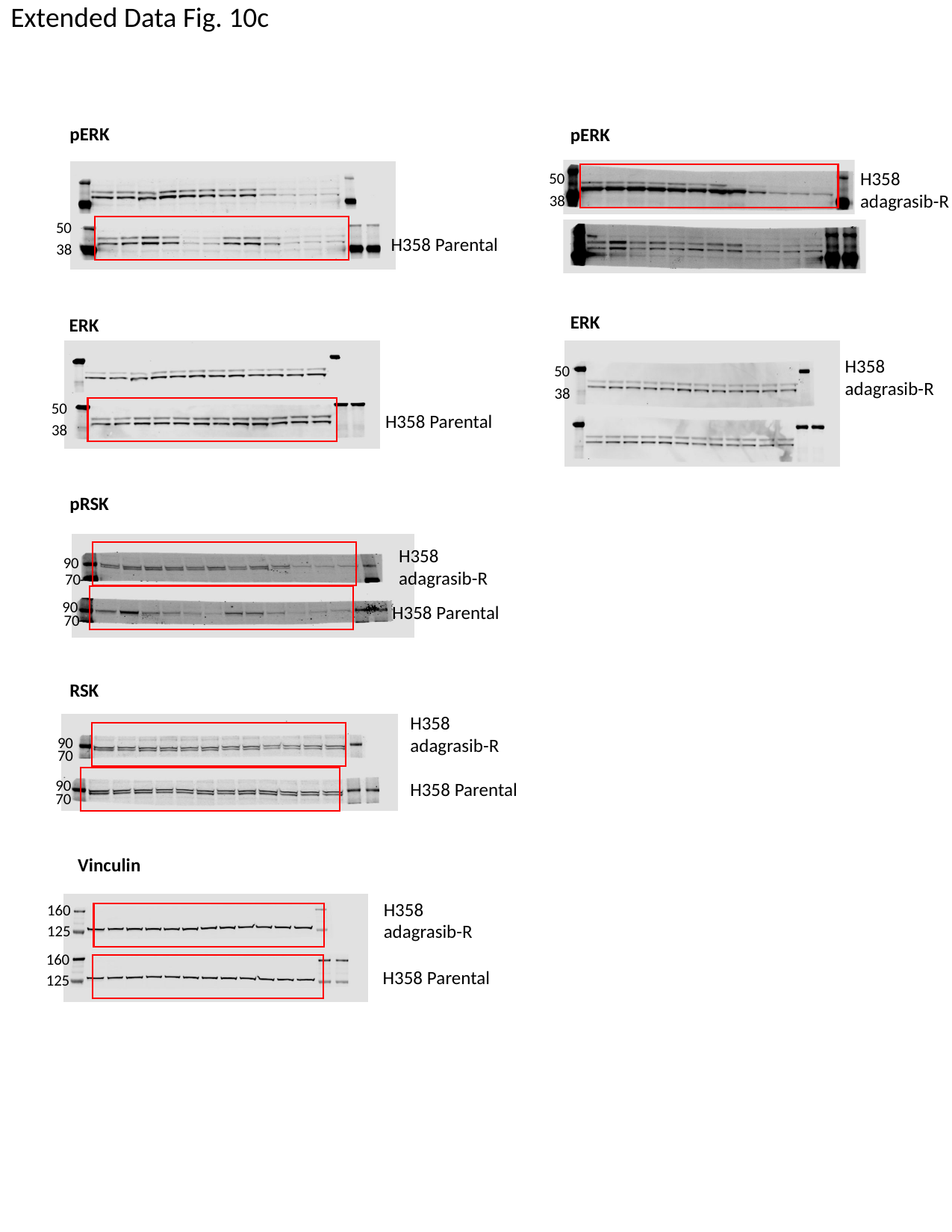

Extended Data Fig. 10c
pERK
pERK
H358
adagrasib-R
50
38
50
H358 Parental
38
ERK
ERK
H358
adagrasib-R
50
38
50
H358 Parental
38
pRSK
H358
adagrasib-R
90
70
90
H358 Parental
70
RSK
H358
adagrasib-R
90
70
90
H358 Parental
70
Vinculin
H358
adagrasib-R
160
125
160
H358 Parental
125
